# Using Electrochemistry to Benchmark, Understand, and Develop Noble Metal Nanoparticle Syntheses

**DOI:** 10.1021/acsnanoscienceau.5c00051

**Published:** 2025-07-18

**Authors:** Gabriel C. Halford, Sebastian Hertle, Harikrishnan N. Nambiar, Michelle L. Personick

**Affiliations:** Department of Chemistry, 2358 University of Virginia, Charlottesville, Virginia 22904, United States

**Keywords:** metal nanoparticles, nanoparticle synthesis, shape control, electrochemistry, open-circuit measurement, benchmarking, electrodeposition

## Abstract

The complex chemical nature of metal nanoparticle synthesis presents obstacles for the mechanistic understanding of nanoparticle growth and predictive synthesis design, despite significant progress in this area. Real-time characterization of the chemical processes that take place throughout nanoparticle growth will enable progress toward addressing outstanding challenges in metal nanoparticle synthesis, such as mitigating synthetic reproducibility issues, defining chemical mechanisms that direct nanoparticle growth, and designing synthetic conditions for previously unachievable combinations of nanoparticle shape and composition. In this Perspective, we present open-circuit potential (OCP) measurements as an in situ, real-time method for characterizing chemical changes during nanoparticle growth and discuss the method’s strengths in comparison to and in combination with other characterization techniques. We propose the use of OCP measurements as benchmarks for troubleshooting irreproducibility and streamlining synthetic optimization. Finally, we explore possibilities for using the increased parameter space accessible by electrodeposition to accelerate the development of shape-selective nanoparticle syntheses.

## Graphical abstract

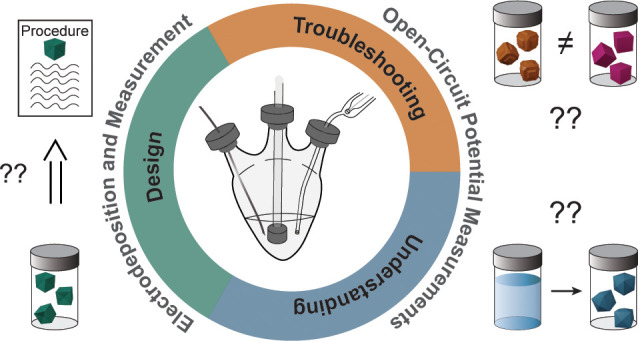

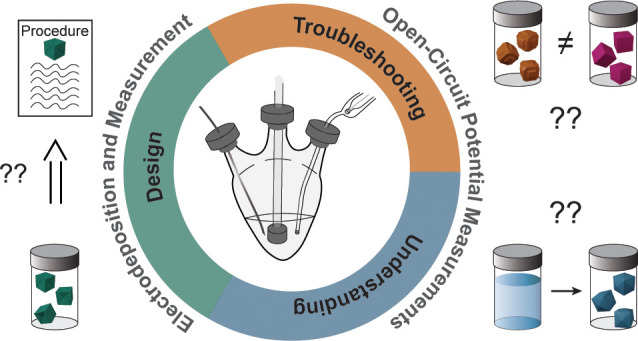

## 1 Introduction

The considerable importance of shape and surface structure in controlling the chemical and physical properties of nanoscale materials has motivated significant research into methods for producing well-defined polyhedral nanoparticles bound by surface facets with distinct atomic arrangements. Such surfaces range from highly coordinated “low-index” facets to surfaces composed of many atomic steps with a high density of undercoordinated surface atoms. Processes that take place primarily on the nanoparticles’ surface—such as catalysis—are most strongly influenced by surface atomic arrangement, although not all catalytic transformations are structure-sensitive.^1–4^ Other characteristics of nanoparticles—such as their optical properties or biological interactions—can originate from the overall shape of the particle as well as the structure and functionalization of the surface.^5–10^

Commonly, new syntheses for shape-controlled nanoparticles are developed using a combinatorial approach, in which the conditions of a previously published synthesis (pH, concentration of additives or reagents, temperature, etc.) are systematically varied. The success of this approach has been amplified by the availability of moderate temperature, aqueous synthesis options for colloidal noble metal nanoparticles. Many syntheses can be set up simultaneously, allowing for the fast screening of reaction conditions. So far, a vast library of syntheses for nanomaterials with different shapes, sizes, and facets has been developed using this combinatorial approach, including simple low-index faceted shapes as well as more complex twinned and high-index faceted shapes.^11–15^ Iterative, combinatorial approaches have been especially successful for nanoparticles composed of gold (Au) and palladium (Pd), in large part because the specific mild reaction conditions used to synthesize some of the earliest examples of shaped nanoparticles are particularly amenable to these compositions, due to the relatively high reduction potential of their precursor complexes.^15–20^

Monitoring the progress of nanoparticle growth during synthesis is not generally necessary to ensure product quality and can be time-prohibitive if many nanoparticle growth reactions are set up at one time, as is common. Therefore, observation and characterization of the resulting nanoparticles is typically only conducted following the completion of the reaction. Because this analysis is done at the end of the synthesis, common characterization methods focus on the properties and attributes of the nanoparticle products, rather than the chemical characteristics of the nanoparticle growth environment. This approach to characterization unintentionally creates a situation where nanoparticle synthesis development can sometimes be a “black box,” resulting in problems during the reproduction of nanoparticle syntheses if, for instance, impurities are unknowingly introduced or omitted through the reagents used.

The combinatorial approach to synthesis design is common by necessity due to the complex nature of the chemistry of colloidal nanoparticle growth solutions. While nanoparticle synthesis is fundamentally a redox process between metal ions and a chemical reducing agent, it is challenging to model using standard reduction potentials because of the nonstandard conditions used (pH ≠ 0; temperature ≠ 25 °C; concentrations ≠ 1 M) and due to the presence of oxygen in most published synthetic conditions. Further, the use of capping agents and other rate- and shape-directing additives is standard for even simple nanoparticle synthesis routes. These additional reagents greatly complicate specific chemical understanding of redox reactions during nanoparticle growth and introduce important interfacial processes. Establishing detailed chemical mechanisms is particularly challenging due to the multiple possible roles of many common reagents in growth solutions. For instance, halide ions can act as selective or nonspecific surface passivators, facilitate oxidative etching, shift the reduction potential of the metal salt precursor through ligand exchange, or catalyze metal ion reduction.^21–23^ This makes it difficult to assign a particular chemical mechanism to a growth solution component. Additionally, concentrations of reactants and dissolved oxygen are not constant over time, leading to complexities with steady-state attempts at interpreting solution chemistry.

Building robust and predictive mechanistic principles for colloidal nanoparticle growth has long been a goal of the metal nanoparticle research community. Significant progress has been made toward understanding nanoparticle growth mechanisms using a number of approaches that correlate physical changes of the nanoparticles during or after growth with changes in initial growth solution chemistry.^22,24–32^ However, there are still many unknowns in the understanding of even some of the best characterized systems, especially in the growth of nanoparticles composed of metals other than Au and Pd. The development of additional easy-to-use, in situ chemical characterization methods will enable further understanding of reaction chemistry in nanoparticle synthesis to meet the grand challenge of establishing a comprehensive framework for predictive design of nanoparticle growth.

This Perspective highlights recent advances and future opportunities in the use of electroanalytical methods to increase understanding of colloidal nanoparticle synthesis, as well as in the use of electrodeposition to develop new syntheses for shaped metal nanoparticles (Figure 1). Specifically, we discuss how open-circuit potential (OCP) measurements meet the need identified above for an in situ, real-time chemical measurement of nanoparticle growth. These measurements can be used to enhance synthetic reproducibility and streamline synthetic optimization through benchmarking of the real-time chemistry (mixed solution potential) during colloidal nanoparticle growth. Additionally, we analyze the strengths and limitations of other in situ and time-resolved methods for characterizing nanoparticle growth to provide context for how OCP measurements can complement these techniques and overcome these limitations to achieve better understanding of growth mechanisms through real-time measurements of the changing chemical environment during synthesis. Finally, we describe how the increased parameter space and synthetic flexibility accessible by electrodeposition can be used in combination with electrochemical measurements of growth to accelerate synthesis design and discovery.

**Figure 1:**
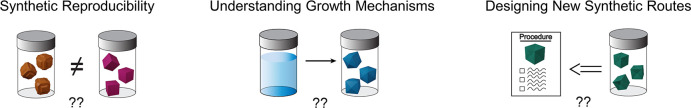
Overview of outstanding problems and questions in metal nanoparticle synthesis that can be addressed via electroanalytical methods (such as open-circuit potential (OCP) measurements), electrodeposition synthesis, or a combination of both.

## 2 Improving Synthesis Reporting and Reproducibility with Benchmark Measurements

### 2.1 Present Practices in Reporting of Nanoparticle Syntheses

To reproduce a published synthesis for metal nanoparticles, one needs an experimental protocol for the synthesis as well as analytical tools to investigate whether the synthesis has been replicated successfully. The literature typically reports experimental synthetic protocols with an explicit focus on the amount of each reagent added and the sequence of addition, as well as the conditions (temperature, pressure, and reaction time) for each reaction step. These are all well-defined synthesis parameters and are, in most cases, easily reproduced. Additionally, the purity and source of the chemical reagents (e.g., sodium tetrachloropalladate­(II) (Na_2_PdCl_4_, ≥99.99% trace metal basis) Sigma-Aldrich), the purity of water used (e.g., 18.2 MΩ resistivity), and the cleaning protocol for glassware (e.g., with aqua regia (1:3 ratio of concentrated HNO_3_: concentrated HCl; **caution: strong acid**)) are commonly reported. Most publications also report a detailed physical characterization of the synthesized nanoparticles at the end of the reaction and, less commonly, throughout the synthesis.

Prevalent methods for characterization of nanoparticles include scanning electron microscopy (SEM) and transmission electron microscopy (TEM), which provide information about the size, shape, composition, and dispersity of the product nanoparticles. Additionally, spectroscopic methods such as ultraviolet–visible spectroscopy (UV–vis) are widely used to characterize shape, size, and uniformity of nanoparticles whose optical properties are structure-sensitive, such as plasmonic nanoparticles. In the case of multimetallic nanoparticles, electronic and X-ray spectroscopies, including energy-dispersive X-ray spectroscopy (EDS) and X-ray photoelectron spectroscopy (XPS), are used to characterize the bulk and surface composition of the nanoparticles, respectively. Additional characterization techniques like atomic force microscopy (AFM), X-ray absorption spectroscopy (XAS), and electron diffraction methods are also used throughout the literature to complement the above techniques.^33^

### 2.2 Challenges for Reproducibility Caused by Present Practices in Reporting of Syntheses

However, a pervasive problem in nanoparticle synthesis is a sporadic lack of reproducibility of published syntheses between—or even within—laboratories,^34–36^ meaning that while the reaction protocol has been accurately followed, the resulting nanoparticles exhibit a different size, dispersity, or morphology than the reported ones. Reproducibility issues can also cause significant challenges for scaling up syntheses of nanoparticles for use in specialized applications. There are multiple underlying reasons for these reproducibility issues. One possible contributing factor is the lack of precise definitions for certain terms used in reaction protocols, such as “vigorously stirred,” “overnight,” “swirl,” or “until completion,” which can be interpreted differently by each researcher reproducing the procedure. Solely reporting the sequence of reagent addition instead of discrete time points for the addition of these chemicals can influence the morphology of the resulting nanoparticles as well. Moreover, the manufacturer and part number of vials, flasks, or stir bars are often not detailed, which can lead to differences in the size, shape, and quality of the reproduced nanoparticles during synthesis replication.

An even more consequential and widely investigated problem for synthetic reproducibility is the role of differing impurity levels in common chemical reagents.^34–36^ These impurities can be beneficial and/or detrimental to shape formation, sometimes in a concentration-dependent manner. The shape-determining role of such impurities has been extensively studied, including for reagents such as cetyltrimethylammonium bromide (surfactant, capping agent),^37–40^ polyvinylpyrrolidone (capping agent, reducing agent),^41–43^ poly­(vinyl alcohol) (capping agent),^44^ oleylamine (capping agent, reducing agent),^45,46^ and ethylene glycol (solvent, reducing agent).^47–49^ In some cases, the impurities are inorganic ions, such as iodide, chloride, or iron.^38–40,49^ For other reagents, the impurities are organic contaminants, such as sodium acetate, acetone, or methanol.^39,44^ Even differences in the structure of the desired reagent can act as impurities, such as the presence of the trans isomer (elaidylamine) as an impurity in oleylamine^45,46^ or differences in the end group functionalization of polyvinylpyrrolidone.^43^ These impurities can influence nanoparticle growth through multiple mechanisms, including altering metal ion reduction kinetics, passivating surfaces, and/or facilitating etching. Complicating the reproducibility of nanoparticle synthesis further is that lot-to-lot differences between chemicals from the same manufacturers can exist, influencing the product of a nanoparticle synthesis even if the same manufacturer and product line are used.^39,40^

The present method of reporting nanoparticle syntheses—which provides only reaction steps and the detailed physical characterization of the nanoparticles—is often insufficient when troubleshooting reproducibility issues in general, as well as for identifying the influence of impurities. While discussions of proposed reaction mechanisms can hint at reasons for reproducibility issues, the absence of real-time information about the chemical changes occurring in the growth solution of the reported reaction over time makes the resolution of reproducibility issues a time and resource-intensive iterative task.^35,39,41,42,46^ Reporting, by default, a benchmark reference measurement of the chemical growth environment of the reaction over the course of the synthesis to complement the physical characterization and the reaction protocol that are already reported for nanoparticle syntheses would streamline the resolution of impurity and reproducibility issues that subsequently emerge. To make this feasible, a reproducible, facile, and accessible method of collecting in situ, real-time information about the chemical reaction environment is required.

### 2.3 Open-Circuit Potential Measurements as a Benchmark of Reaction Chemistry

OCP measurements represent a promising approach for generating benchmark measurements of the chemical reaction environment behind a successful nanoparticle synthesis. While the many competing processes in even simple nanoparticle growth solutions make them mechanistically complex, the overall chemistry of nanoparticle growth can be monitored via measurement of the mixed potential of the growth solution during the particle growth process. Time-resolved measurement of the solution potential during nanoparticle growth can be achieved through OCP measurements of the growth solution, taken in an electrochemical cell that includes a working electrode (for example, a glassy carbon electrode) and a reference electrode (such as silver/silver chloride (Ag/AgCl) or mercury/mercurous sulfate (Hg/HgSO_4_)). Though it is not strictly required for OCP measurements, the inclusion of a counter electrode (for instance, a platinum (Pt) wire) to create a three-electrode cell is common. The electrodes are introduced to a colloidal nanoparticle growth solution prior to the initiation of growth by a chemical reducing agent, and the solution potential is measured over time (Figure 2).

**Figure 2:**
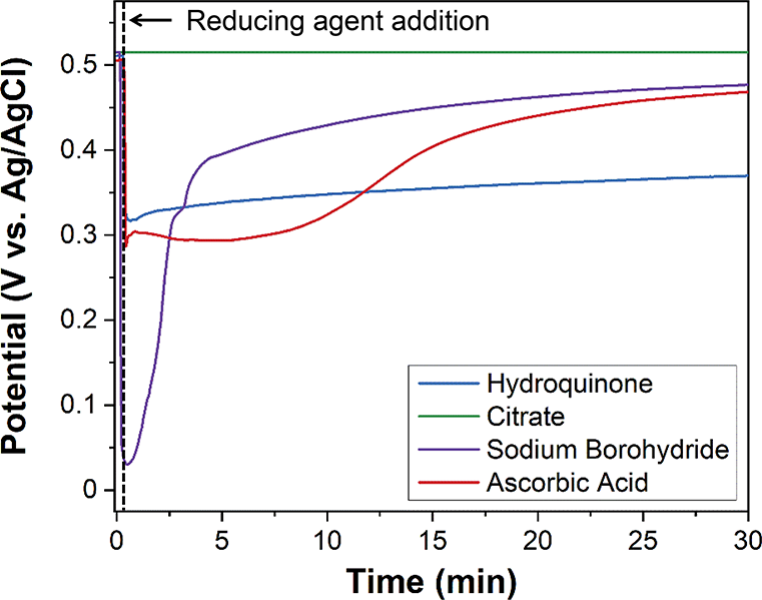
Example OCP measurements illustrating real-time mixed potential responses, with the time point of addition of chemical reducing agent noted with a dotted line. The measurements shown are for colloidal growth solutions in a halide-free surfactant (cetyltrimethylammonium hydrogen sulfate) containing 0.5 mL of 10 mM H_2_PdCl_4_, with a 2-fold stoichiometric excess of reducing agent added after initiating measurement (0.1 mL of 100 mM hydroquinone, l-ascorbic acid, hydroxylamine hydrochloride, or trisodium citrate or 0.025 mL of 100 mM sodium borohydride). Adapted with permission from ref 50. Copyright 2020 American Chemical Society.

The OCP is the potential measured between the working electrode and the reference electrode when no applied current is flowing.^51,52^ At any given potential at any given time, the working electrode passes a total current that is the sum of all individual currents arising from the anodic and cathodic half reactions taking place there. At the OCP, the net current is zero, since there is no external current flow. Thus, the sum of the cathodic currents and the sum of the anodic currents have equal magnitudes and opposite signs. If the individual component currents vary over time, such as when nonequilibrium chemical reactions are occurring, the zero-current point (and, therefore, the measured OCP) will drift.^51^

When the cathodic and anodic currents at the working electrode are dominated by a single redox couple, the OCP is equal to the equilibrium potential of the redox couple determined by the Nernst equation. However, when the OCP is controlled kinetically by two or more half reactions, such as during metal nanoparticle growth, the OCP is not an equilibrium potential, but rather a mixed potential.^53^ When measuring the OCP of a nanoparticle growth solution, contributions to the mixed potential may include metal ion reduction, reducing agent oxidation, dissolved oxygen reduction, electrolyte decomposition, and/or other contributions from other electroactive species such as halide ions or ionic surfactants. These additional contributions to the mixed potential are the reason why the measured OCP has an initial nonzero “background” value before the initiation of metal ion reduction with injection of the chemical reducing agent and seeds.

While the determination of the mixed potential from theory is generally impractical, some general considerations for predicting the effects of chemical and physical parameters on the measured OCP do exist. Solute concentration is known to affect the OCP such that when a redox-active solute is at a millimolar or higher concentration and all other species’ concentrations are order(s) of magnitude lower, the OCP is pushed toward the Nernstian equilibrium potential. When the solute’s concentration is nanomolar or lower, its contribution to the mixed potential becomes negligible and the OCP approaches the “background” value.^51,54^ This concentration effect on OCP is an important consideration for any measurements of especially dilute growth solutions, such as those used for some nanorod syntheses,^55^ as other background contributions to the mixed potential may partially mask the OCP contribution from metal ion reduction. Additionally, significant local pH changes near or at the working electrode, such as those due to metal ion reduction reactions that release protons, can cause rapid changes or oscillatory behavior in the measured OCP.^56^

The mixed potential captured in OCP measurements is representative of all chemical processes (and the physical or interfacial processes perturbing chemical processes) occurring in the growth solution in real time. The reducing environment resulting from this combination of chemical processes influences the kinetics of metal ion reduction and, therefore, the growth pathway and ultimate nanoparticle product morphology. Early examples of OCP measurements of Pd nanoparticle growth suggest that there is a correspondence between the measured strength of the reducing environment (the mixed solution potential)—as well as changes in this solution potential—and the development of particular morphologies for a given system.^39,50^ Because of their ability to record real-time differences in reaction chemistry that directly correlate to particular synthetic outcomes, OCP measurements of solution-phase nanoparticle growth have demonstrated promise as a technique of choice for benchmarking optimal growth conditions (Figure 3A).^39^

**Figure 3:**
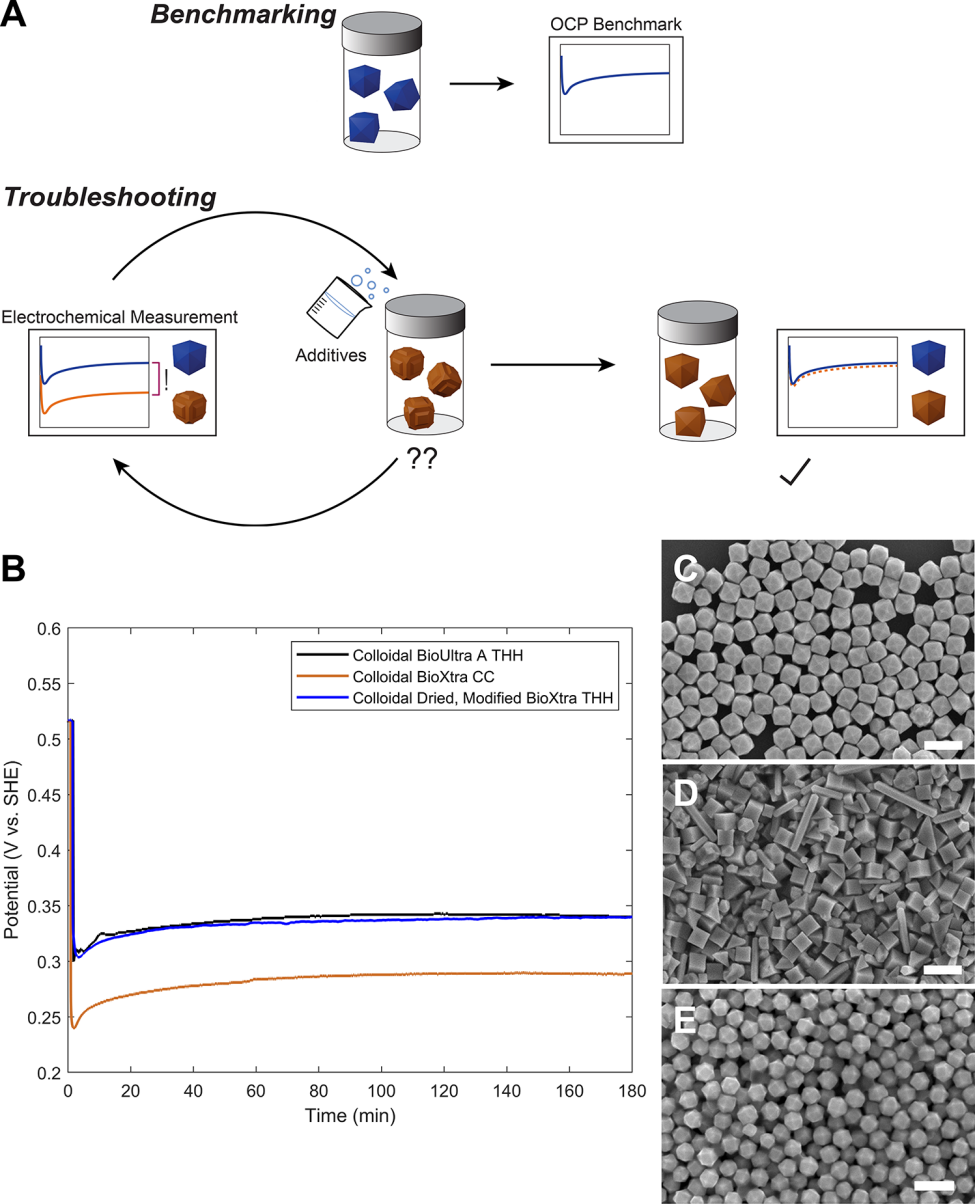
(A) Schematic of the OCP measurement approach for benchmarking nanoparticle synthesis and troubleshooting reproducibility issues. (B) OCP measurements of growth solutions made in as-received BioUltra A CTAB (black) and BioXtra CTAB (orange)—both using the conditions developed for Pd THH growth in BioUltra A CTAB—and a growth solution made in oven-dried BioXtra CTAB with shape-directing additives (blue; 0.58 μM NaI and 230 μL of acetone (0.31 M in the growth solution)). (C–E) SEM images of colloidal Pd THH and CC nanoparticles. (C) Pd THH nanoparticles synthesized in as-received BioUltra A CTAB. (D) Pd CC nanoparticles synthesized in as-received BioXtra CTAB but otherwise under the same conditions as (C). (E) Pd THH nanoparticles synthesized in oven-dried BioXtra CTAB with 0.58 μM of NaI and 230 μL of acetone added to the growth solution. Scale bars: 300 nm. Panels B-E adapted with permission under a Creative Commons CC BY-NC 3.0 license from ref 39. Copyright 2024 Royal Society of Chemistry.

We recently provided proof of concept for this OCP benchmarking approach by using these measurements to inform troubleshooting of reproducibility issues in the synthesis of Pd tetrahexahedra (THH) stemming from the previously mentioned line-to-line and lot-to-lot variability in high-purity commercial cetyltrimethylammonium bromide (CTAB) surfactant.^39^ When Sigma-Aldrich BioUltra CTAB (99.0%, Lot No. BCCC5274; “BioUltra A CTAB”) was used as the surfactant in Pd nanoparticle synthesis, monodisperse THH (99% of particles) were formed (Figure 3C). When another lot number of the same BioUltra CTAB line (99.0%, Lot No. BCCF7530; “BioUltra B CTAB”) or a similarly high-purity line (Sigma-Aldrich BioXtra CTAB (99%, Lot No. SLCJ8356)) was used in reactions with otherwise identical growth conditions, the products were concave cubes and various twinned particles (∼75 and ∼25% of particles, respectively, Figure 3D). A comparison of the benchmark OCP measurement for the successful synthesis of Pd THH in BioUltra A CTAB with OCP measurements for unsuccessful Pd nanoparticle syntheses in the other two CTABs revealed a more strongly reducing mixed solution potential in the nanoparticle growth solutions that produced undesired concave cubes compared to the mixed potential of the successful THH reaction conditions (Figure 3B).

Shape-directing iodide and acetone or methanol impurities were identified in all CTAB varieties via inductively coupled plasma mass spectrometry (ICP-MS) and nuclear magnetic resonance (NMR) spectroscopy, respectively.^39^ “Beneficial,” controlled amounts of both impurities were determined to be necessary for successful Pd THH formation. Following oven drying of the as-received BioUltra B CTAB and BioXtra CTAB powders to eliminate residual organic solvent impurities that were in excess of the ideal amounts, OCP measurements of “modified” reaction solutions—into which controlled small amounts of iodide and acetone or short-chain alcohols were added—showed that the addition of iodide increased the mixed solution potential, shifting it toward the benchmark value, as did the readdition of a smaller concentration of organic solvent. Through modification of the growth solution with these impurity species, it was possible to adjust the measured mixed solution potential of the reaction in BioUltra B CTAB and BioXtra CTAB to match that of the successful Pd THH synthesis in BioUltra A (Figure 3B), and to successfully synthesize the Pd THH using these other types of CTAB (Figure 3E). We hypothesized that these species (iodide and organic solvent) disrupt the surfactant bilayer at the surface of the growing nanoparticle, thus increasing the rate of metal ion reduction and, correspondingly, the rate of consumption of the chemical reducing agent ascorbic acid (AA). Importantly, while the OCP measurement records the solution potential and changes in the solution potential, if surface passivation affects the overall reaction rate, this influence is captured as a component of the measurement.

We next demonstrated that the OCP benchmarking and troubleshooting approach could be used to expedite resolution of reproducibility challenges with new batches of CTAB. OCP measurements of Pd nanoparticle growth solutions made in CTAB from Thermo Scientific (99%) and Tokyo Chemical Industries (TCI; 98.0%, “TCI CTAB”) were compared to the THH-forming BioUltra A CTAB benchmark measurement.^39^ The differences between these measurements and the THH benchmark, in combination with previous measurements demonstrating the relationships between iodide and acetone concentrations and the strength of the reducing environment, were used to select NaI and acetone additive concentrations for the Thermo Scientific and TCI CTAB varieties (again following oven-drying of the CTAB powder). Only one round of additional minor synthetic modification was necessary to achieve OCP traces in good agreement with the BioUltra A CTAB benchmark (Figure 4A) and corresponding successful formation of the THH product morphology (Figure 4B–D). These experiments demonstrated the power and utility of the OCP benchmarking and troubleshooting approach. Importantly, this second set of corrections based on the benchmark required only 3 days, which was much less time-consuming than troubleshooting via trial-and-error variation of synthetic conditions without OCP measurements (which had previously been used to attempt Pd THH optimization for other CTAB sources, taking years).

**Figure 4:**
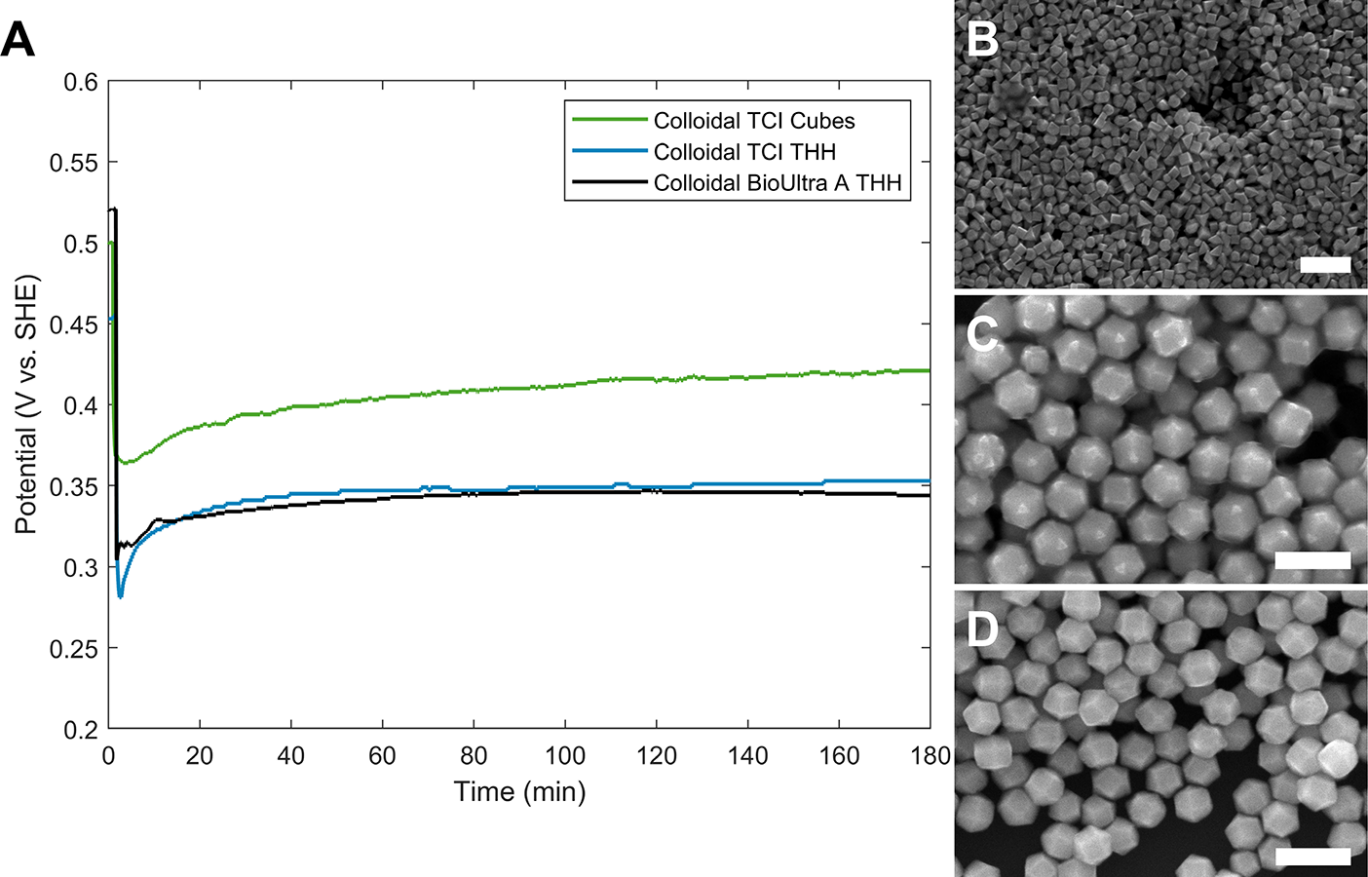
(A) Open-circuit potential (OCP) measurements of growth solutions made in as-received BioUltra A CTAB (black; SEM shown in Figure 3C), as-received TCI CTAB (green)—both using the Pd THH growth conditions developed for BioUltra A CTAB—and in oven-dried TCI CTAB with shape-directing additives (blue; 0.48 μM NaI and 90 μL of acetone (0.12 M in the growth solution)). (B–D) SEM images of colloidal Pd nanoparticles synthesized in TCI CTAB. (B) Pd nanocubes synthesized in as-received TCI CTAB (green OCP trace in (A)). (C) Truncated Pd THH nanoparticles synthesized in oven-dried TCI CTAB with 0.58 μM added NaI and 100 μL added acetone (0.14 M in the growth solution). (D) Pd THH nanoparticles synthesized in oven-dried TCI CTAB with 0.48 μM of NaI and 90 μL of acetone additives (0.12 M acetone in the growth solution; blue OCP trace in (A)). Scale bars: 300 nm. Adapted with permission under a Creative Commons CC BY-NC 3.0 license from ref 39. Copyright 2024 Royal Society of Chemistry.

The real-time nature of OCP measurements and their comparative ease of setup allow benchmark measurements of the growth process to be easily created for all synthetic conditions of interest. These OCP benchmarks, in turn, provide a record of the optimal behavior of a nanoparticle synthesis reaction from a *chemical* perspective *during* the reaction, rather than just observing that the product is correct. Whenever reproducibility issues with a colloidal nanoparticle synthesis are subsequently observed via any commonly used characterization technique, comparison of a new OCP measurement to the benchmark measurement for the reaction of interest can elucidate discrepancies between the relative strength of the reducing environment over time compared to the ideal case. This can then be readily correlated with synthetic parameters affecting the reducing environment, such as the ratio of metal ions to chemical reducing agent or the concentration of halide ions. Troubleshooting would then involve manipulating a relevant synthetic parameter in a direction and with a magnitude guided by the difference from the benchmark measurement.

### 2.4 Experimental Considerations for Open-Circuit Potential Measurements

When obtaining benchmark OCP measurements, appropriate selection of electrodes is key for ensuring measurement accuracy. The working electrode material must be inert under the conditions of the synthesis reaction, as plating of metal salts from the growth solution onto the working electrode will shift the measured potential.^56^ Glassy carbon is an appropriate working electrode material for use with a variety of metals. While the surface area of the working electrode does contribute to the OCP of solutions containing metal nanoparticles when at least one of the electrode dimensions is also at the nanoscale or low microscale (in the case of ultramicroelectrodes),^53,57^ this contribution becomes negligible and electrode area does not need to be considered when larger electrodes are used (as is the case in all studies described in this Perspective). Reference electrode choice also must be informed by the chemistry of the growth solution. Silver/silver chloride (Ag/AgCl) reference electrodes were used for our initial OCP measurements of colloidal Pd particles;^50^ however, we switched to using a mercury/mercurous sulfate reference electrode (Hg/HgSO_4_) for measuring colloidal Pd THH growth solutions that contained NaI as a shape-directing additive^39^ because I^–^ contamination can shift the Ag/AgCl reference potential through formation of an AgI layer on the electrode.^58,59^ A reference electrode other than Ag/AgCl should be chosen in situations where Ag^+^ and/or Cl ^–^ leaching could pose interference, such as syntheses where Ag underpotential deposition controls shape or halide-free syntheses.

High solution resistance can affect OCP measurements, but is typically sufficiently minimized through placement of the working electrode and the bridge tube containing the reference electrode close together (less than 1 cm apart with approximately the same distance between the two from measurement to measurement); a Luggin capillary may also be used in place of a traditional bridge tube to further minimize solution resistance. The use of high-concentration insulating growth solution additives, such as some polymers, could cause sufficient solution resistance to be problematic for OCP measurements; however, we have not observed such issues with decimolar concentrations of C_16_ surfactants or double-tailed C_18_ surfactants (unpublished results). Mass transport limits can affect the OCP,^57^ and mass transport efficiency is commonly controlled in the electrochemistry literature through the use of stirring or a rotating disk electrode (RDE). However, to best mimic the procedure for unmeasured colloidal nanoparticle syntheses, which are often swirled following the addition of all reagents and then left unstirred for the duration of the reaction, we have typically stirred growth solutions during OCP measurements for several seconds following the addition of the last reagent and then turned the stirring off.^39,50^

Other important considerations for making reproducible OCP measurements include cleaning and maintenance of equipment. As is generally the case for studies of metal nanoparticle synthesis, cleaning of glassware with aqua regia between uses (including cleaning of the reference electrode bridge tube) and appropriate storage and fresh preparation of stock solutions are crucial. Polishing of glassy carbon working electrodes with an alumina slurry and subsequent cleaning in an ultrasonic bath are also required after each use; other electrodes may require different polishing and surface activation steps, such as sanding for Ag wire electrodes. We have not found aqua regia cleaning of glassy carbon electrodes between uses to be necessary. Regular maintenance of reference electrodes—refilling of electrolyte solutions, changing of frits, and verification against a “master” reference electrode or calibration solution of a known redox couple, such as ferrocene/ferrocenium—is also crucial for ensuring accurate measurements.

Care should also be taken to make sure that the morphological result of the synthesis is not altered by the experimental setup of the measurement. For example, moving a synthesis from a scintillation vial to a beaker or specialty electrochemistry glassware may induce problems for syntheses with glassware sensitivities (common for Ag nanoparticle synthesis), and the choice of an acidic salt bridge electrolyte such as sulfuric acid to fill the reference electrode bridge tube may alter, for instance, a highly pH-sensitive synthesis. A best practice is to characterize and “verify” all measured products through orthogonal techniques such as electron microscopy or optical spectroscopy until a robust protocol for OCP measurement is established for a particular synthetic system via selection of compatible glassware and electrodes (Figure 5). Once a protocol has been established, experimental setup is facile and OCP measurements themselves generally show good batch-to-batch, day-to-day, and researcher-to-researcher reproducibility.

**Figure 5:**
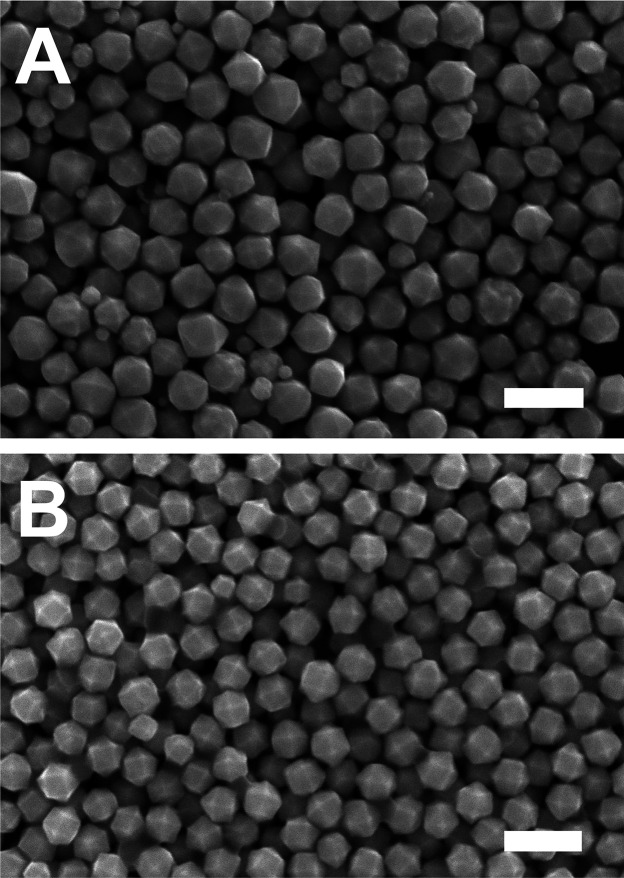
SEM images of colloidal Pd THH particles which were (A) unmeasured and undisturbed and (B) subject to OCP measurements throughout growth. Both growth solutions were prepared with oven-dried BioXtra CTAB with additives: 0.58 μM NaI and 230 μL of acetone (0.31 M in the growth solution). Scale bars: 300 nm. Panel B adapted with permission under a Creative Commons CC BY-NC 3.0 license from ref 39. Copyright 2024 Royal Society of Chemistry.

## 3 Understanding Nanoparticle Growth Mechanisms

Beyond issues of synthetic reproducibility, new insights into specific chemical mechanisms of nanoparticle growth are necessary for a more predictive approach to synthesis design. While comparison of OCP measurements for benchmarking and troubleshooting purposes does not necessarily require detailed deconvolution of the specific individual processes contributing to the overall mixed solution potential, the mixed solution potential contains a wealth of additional chemical information that can be further analyzed. OCP measurements capture information related to metal ion reduction rate and reducing agent oxidation, as well as surface interactions and diffusion if they affect the redox process. Work to understand how to extract these parameters and mechanistic information easily from an OCP measurement, using orthogonal analytical techniques to probe each one, is ongoing.

A particular opportunity afforded by the use of OCP measurements is the ability to observe chemical changes in the growth solution directly, rather than studying physical changes to nanoparticles during or following growth. In this section, we will first summarize progress made with other frequently used techniques for in situ and time-resolved study of nanoparticle growth mechanisms. Section 3.1 provides detailed contextual background on the strengths and limitations of these other techniques: electron microscopy, UV–vis spectroscopy, inductively coupled plasma spectroscopies, and NMR spectroscopy. A reader who has a high degree of familiarity with the nuances of each technique may wish to skip directly to Section 3.2. We will then highlight how time-resolved, in situ solution potential measurements can complement and fill gaps in understanding left by these other analysis techniques.

### 3.1 Common Approaches to the Time-Resolved Study of Metal Nanoparticle Growth

#### Electron Microscopy

Electron microscopy techniques such as SEM and TEM are some of the most common physical characterization methods for metal nanoparticles, each with unique benefits: SEM has its advantages in capturing three-dimensional structural information and TEM provides increased resolution, down to the atomic scale. Most often, both SEM and TEM imaging are used for the characterization of nanoparticle products following the conclusion of growth. However, tracking the size and shape development of nanoparticles during growth is feasible through point-in-time imaging with SEM as well as TEM.^22,27,60–63^ In this method, nanoparticle growth is stopped through chemical or mechanical means at a defined time point of the reaction. A near-instantaneous stop in reaction progress can be achieved by a chemical quenching agent, often the chelating agent bis­(p-sulfonatophenyl)­phenylphosphine dihydrate potassium salt (BSPP) (discussed further in the ICP-OES/MS section),^22,27,60^ although cystine has also been used.^61^ Unfortunately, the strong adsorption of BSPP on the surface of metal nanoparticles often leads to charging-related image artifacts and a decrease in image quality.^60^ Instead of chemically quenching the reaction, an aliquot of the growth solution can be centrifuged for several minutes at a specific time point to isolate nanoparticles from the supernatant containing the reducing agent and unreacted metal ions, stopping the further growth of the nanoparticles. However, the minutes-long centrifuging step in this method limits time resolution.

Electron microscopy additionally allows for the further characterization of the structure and composition of nanoparticle growth intermediates using complementary characterization techniques like EDS (SEM and TEM), electron energy loss spectroscopy (EELS; TEM only) and electron diffraction (TEM only).^62–65^ For example, Ahn et al. paired point-in-time TEM imaging with EDS mapping to study Au, Pd, Ag, and multimetallic nanostructures throughout their growth.^61^ Scanning transmission electron microscopy (STEM) can also provide high-resolution characterization of shape development and elemental distribution,^61,63–66^ such as the tracking of the development of single-crystalline Au seeds into 20-fold twinned Ag–Au icosahedra through sequential growth of Ag tetrahedra onto the Au seeds.^62^

In addition to point-in-time electron microscopy methods, in situ TEM techniques have become a significant tool for studying nanoparticle growth.^67–69^ These techniques enable the observation of growth, dissolution, and shape transformations in real space and real time under vacuum, liquid, and gas environments.^67^ Liquid phase TEM in particular has been utilized to investigate both classical and nonclassical nucleation and growth pathways, as well as the self-assembly of nanoparticles.^67,68,70,71^ Early examples of liquid phase TEM involved the time-resolved study of copper (Cu) nucleation and growth during electrodeposition,^72,73^ while additional examples have probed Ag–Pd and Ag–Au galvanic replacement reactions on Ag nanoparticle templates,^74,75^ and the development of core–shell bimetallic structures.^76,77^ An advantage of in situ imaging in a liquid cell is the capture of much smaller time intervals than point-in-time imaging following reaction quenching. This opens up the investigation of seed formation and growth dynamics, which are critical for establishing nanoparticle structure at early time points. Liao et al. used a liquid cell TEM in conjunction with a fast-detection camera to track the facet development of Pt nanocubes during the first seconds of growth,^78^ and Gao et al. later followed up this work with liquid cell observation of the development of high-index facets from Pt cubes following electron beam irradiation.^79^ Time-lapse liquid cell TEM imaging has also been used to understand nanoparticle growth by monitoring the dissolution of nanoparticles through etching.^69,80,81^ Ye et al. studied the oxidative etching of Au nanorods, nanocubes, and rhombic dodecahedral nanoparticles in a redox environment in a liquid cell, using time-lapse TEM imaging to track the shape of intermediates throughout the course of dissolution.^82^ These time-lapse images revealed tetrahexahedral structures as an intermediate during the nonequilibrium etching process of multiple Au nanocrystal shapes (Figure 6).

**Figure 6:**
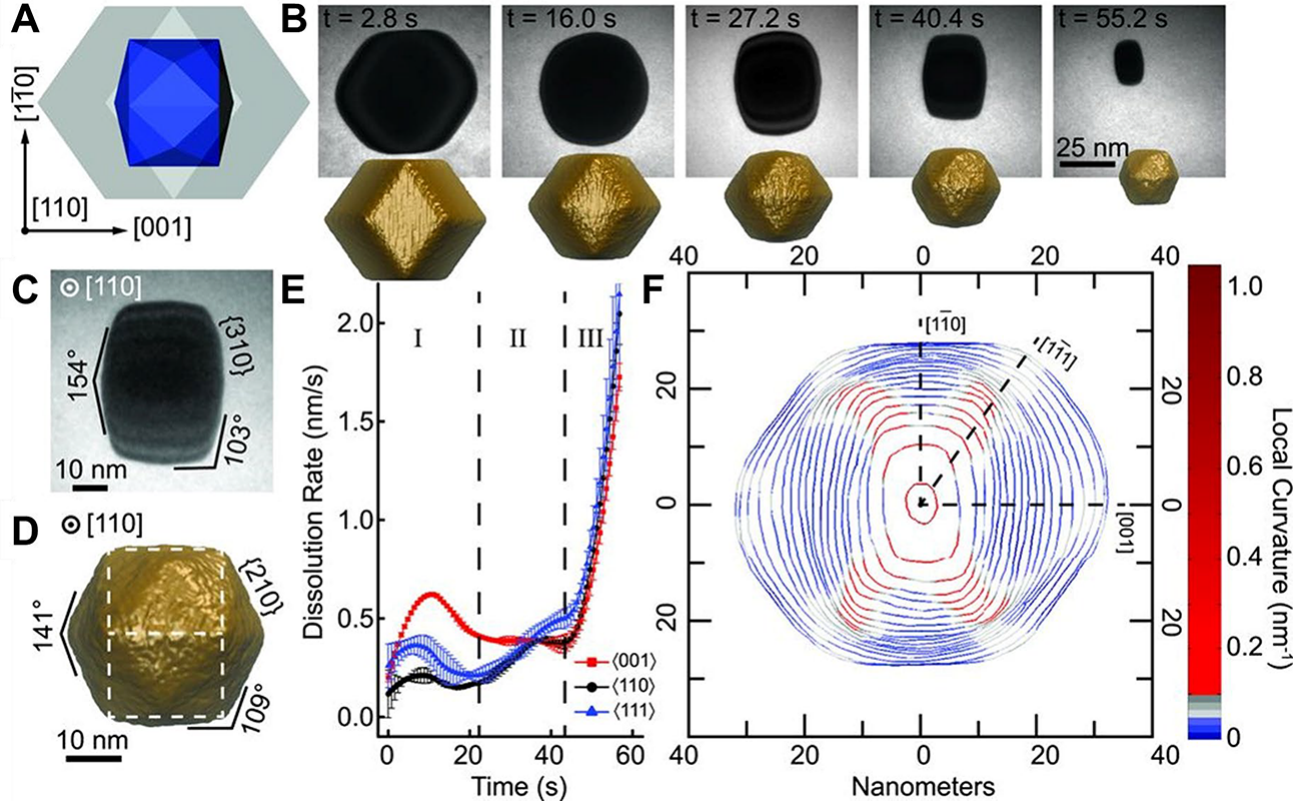
Transition of a rhombic dodecahedral Au nanoparticle to a THH shape during nonequilibrium etching by a TEM beam. (A) Model of a rhombic dodecahedron (gray) with intermediate THH shown internally (blue). (B) Time-lapse TEM images during etching and corresponding snapshots from Monte Carlo simulations of the etching process. THH intermediates with labeled zone axis and calculated {*hk*0} facet from (C) experiment and (D) simulation. (E) Crystallographic particle dissolution rates extracted from contour plots, averaged over several symmetric directions. (F) Time-domain contour plots, showing contour every 1.6 s during etching, with relevant crystallographic directions labeled as dotted lines. Adapted with permission from ref 82. Copyright 2016 American Association for the Advancement of Science.

#### UV–Visible Spectroscopy

Optical spectroscopy is an additional method often used to study the time-resolved growth of nanoparticles. The first of the two methods for monitoring nanoparticle growth by UV–vis is based on measuring a decrease in the concentration of the metal precursor over time. A prerequisite to accurately measuring the precursor concentration using UV–vis is ensuring that the absorption peak of the precursor does not overlap with the adsorption peaks of other chemicals in the growth solution or the localized surface plasmon resonance (LSPR) peak from the forming nanoparticles in the case of plasmonic materials. To achieve this, the nanoparticles are often separated from the growth solution via centrifugation before determining the precursor concentration, which can be difficult at early time points of the reaction due to the small size of the particles.^83,84^ Additionally, the precursor should not undergo hydrolysis if dilution of the growth solution is required to allow for reproducible UV–vis measurements.^83,84^

Time-resolved precursor concentration studies using UV–vis have been extensively utilized to investigate the mechanism of Pd nanoparticle growth because the maximum absorbance of Pd precursors at a specific wavelength in the UV–vis spectra shows a linear dependence on the precursor concentration.^83–88^ For example, Yang et al. measured the change in Pd precursor concentration over time during the nanoparticle growth reaction in a study of the influence of reduction kinetics on the precursor reduction pathway in Pd nanoparticle synthesis.^85^ As the kinetics of precursor reduction are directly tied to nanoparticle growth outcomes, this allowed for quantitative analysis of reaction kinetics in Pd nanoparticle growth and identification of differences in the reduction pathway between a PdCl_4_^2–^ and a PdBr_4_^2–^ precursor. Precursor concentration studies in nanoparticle growth have additionally been carried out for Au nanoparticles, although a rapid ligand exchange and the presence of multiple intermediates complicate the quantitative interpretation of UV–vis kinetics data for both Au and Pt precursors, limiting the widespread use of this approach.^89,90^

In a second method, the growth of plasmonic nanoparticles is investigated directly by analyzing the change in the LSPR peak of the nanoparticles over time. The position of the absorbance maxima of the LSPR is characteristic of the shape, size, and composition of a nanoparticle, allowing one to monitor the growth of nanoparticles of a specific shape over time from UV–vis. Information about the development of the size of the nanoparticles can be gathered, as an increase in particle size leads to a shift of the longitudinal LSPR to longer wavelengths (red shift). In most cases, the information obtained from the UV–vis spectra is solely qualitative, giving information about the concentration, shape, and size of the particles as well as trends in these characteristics.^91–94^ In a recent example, our research group used time-resolved UV–vis to study the mechanism of a visible light-induced interconversion of Ag nanoprisms to icosahedra.^95^ The gradual onset of the characteristic absorbance peaks of Ag icosahedra at ∼450 nm, with the simultaneous decrease of the characteristic peak of the Ag prisms in the near-infrared (NIR) throughout the course of the reaction, indicated an interconversion of the prisms into icosahedra at illumination with light of a specific wavelength. (Figure 7A). This interconversion mechanism was further supported by point-in-time SEM imaging, which additionally indicated a homogeneous nucleation of icosahedral seeds from residual Ag^+^ with subsequent growth of these icosahedra utilizing the oxidative dissolution of the prisms as a source of Ag^+^ (Figure 7B–D).^95^

**Figure 7:**
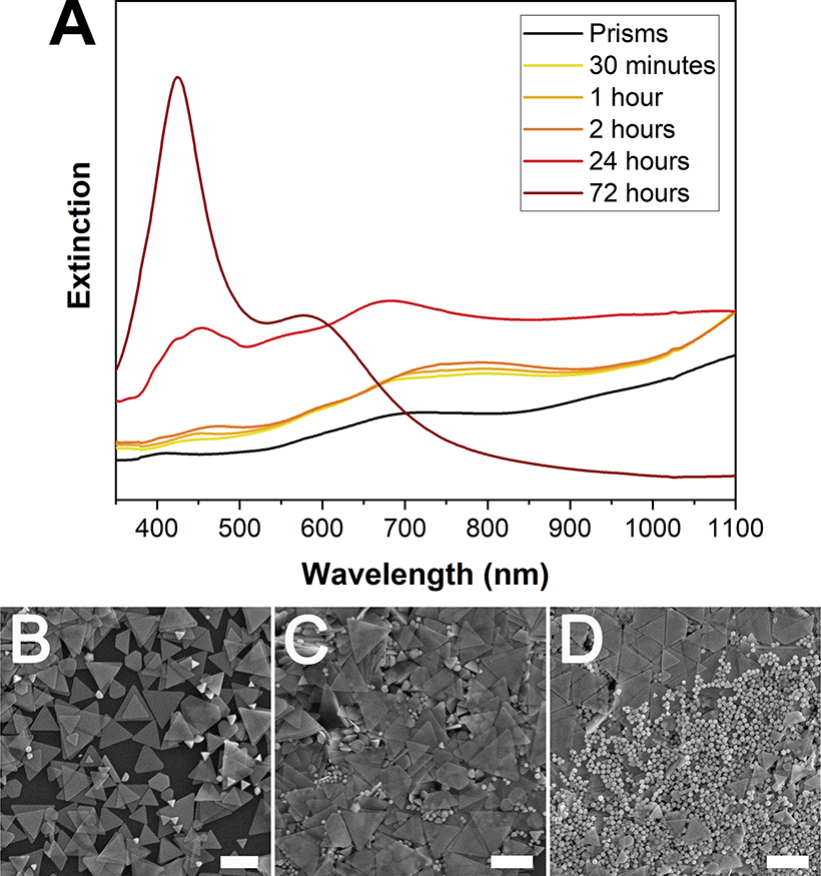
(A) Point-in-time UV–vis extinction spectra of the growth solution at discrete time points during the prism to icosahedra transformation under 400 nm illumination. (B–D) Point-in-time SEM images of Ag prisms during 400 nm irradiation-induced reconfiguration into icosahedra. Images show particles following 400 nm irradiation for (B) 30 min, (C) 60 min, and (D) 120 min. Scale bars: 500 nm. Adapted with permission from ref 95. Copyright 2023 American Chemical Society.

In an additional recent study, UV–vis extinction spectroscopy was used by Simon et al. to monitor the growth of Au, Au/M bimetallic (M = Cd, Fe, Co, Ni, Cu, Ag, Pt, or Pd), and Ag/Pt nanoparticles produced using a molecular photosensitizer as the reducing agent.^96^ In combination with point-in-time TEM imaging, the optical density (O.D.) derived from the UV–vis spectra (a qualitative method for tracking nanoparticle concentration) confirmed that the nanoparticles produced by photocatalytic reduction were continuously nucleated throughout the reaction under sustained illumination, while all particle growth was quenched in the dark. This contrasts with what is typically observed in homogeneously nucleated metal nanoparticle syntheses, where nucleation commonly occurs in a single burst early in the reaction.

LSPR peaks in UV–vis spectra can additionally be studied quantitatively through a fit of Gaussian functions to the spectra.^93,97^ This method was successfully used by Wang et al. in the study of the shape evolution of Ag nanodecahedra and nanoprisms, where different growth mechanisms of Ag nanoparticles were investigated by extracting the time-resolved contributions to the overall UV–vis absorbance resulting from Ag (1) seeds, (2) prisms, and (3) decahedra.^93^ Furthermore, this method allowed the authors to obtain Arrhenius plots of rate vs temperature at different irradiation wavelengths by tracking the seed concentration, which was the Ag^+^ source in this reaction.^93^

#### Inductively-Coupled Plasma Optical Emission Spectroscopy/Mass Spectrometry

Inductively coupled plasma (ICP) spectroscopic methods, including inductively coupled plasma optical emission spectroscopy (ICP-OES) and inductively coupled plasma mass spectrometry (ICP-MS), are commonly used to quantify the concentration of elements of interest. ICP techniques, particularly the more sensitive ICP-MS, have been used to quantify the concentration of elemental impurities in reagents such as CTAB.^38–40^ In addition, quantification of unreacted metal ions in solution and reduced metal incorporated into nanoparticles have both been used as a basis for conducting point-in-time experiments for probing metal ion reduction kinetics for both monometallic and multimetallic nanoparticles.^21,22,28,60,92,98^

The procedure for conducting time-resolved ICP measurements typically involves quenching metal ion reduction in an aliquot of the nanoparticle growth solution at each time point of interest using a chelating agent, commonly BSPP. BSPP was initially selected due to its affinity for chelating Ag and Au ions, but has since proven successful for additional metals.^21,22,28,60,92,98^ The quenched aliquot of growth solution is then centrifuged, and the supernatant (containing BSPP-chelated metal ions) is separated from reduced metal solids. For analysis of the reduced metal solids, the isolated pellets of nanoparticles are then washed, digested in aqua regia, and diluted to an appropriate concentration for ICP-OES or ICP-MS analysis. Supernatant samples typically only require dilution before analysis. The concentration of the element of interest obtained by ICP spectroscopy is representative of the concentration of unreacted, unreduced metal ions (for a supernatant sample) or reduced metal ions incorporated into nanoparticles (from a digested solid sample).

Initially developed for characterizing the kinetics of Ag nanoparticle growth,^92^ time-resolved ICP spectroscopy has since been used to characterize the rate of metal ion reduction for a wide variety of nanoparticle compositions.^21,22,28,30,60,98^ This initial example and a subset of other uses of the technique for understanding Ag nanoparticle growth measured the concentration of residual unreacted metal precursor ions (i.e., Ag^+^; supernatant samples) in solution over time.^92,99–101^ However, this specific approach can be challenging for other synthetic systems that involve large surfactant or capping molecules that can foul or contaminate ICP spectrometers and, as a result, most subsequent examples have instead tracked the amount of reduced metal over time (digested solid samples).^21,22,28,30,60,98^

The as-determined concentrations of metal at different time points are used to generate a kinetic curve based on either the depletion of the metal precursor ion or the increase in the amount of reduced metal over time. Comparison of this data across multiple reaction conditions (i.e., different concentrations of additives) can give qualitative insight into differences in particle growth rate and extent of reaction for different syntheses. This approach has, for instance, been used by our research group to demonstrate the catalysis of metal ion reduction by low concentrations of halide additives.^21^ Point-in-time electron microscopy images are often correlated with time-resolved ICP data. For example, 3 μM I^–^ was found to be necessary for the growth of Au–Pd tetradecapod nanoparticles, while a rhombic dodecahedral shape formed in the absence of I^–^ (Figure 8A,B).^60^ The ICP-OES kinetics data showed that the rates of Pd ion reduction with and without iodide diverged at about 15 min, while the rates of Au ion reduction remained the same (Figure 8C,D). Time-resolved SEM imaging of tetradecapod formation revealed that, prior to 14 min of growth, the particles had a rhombic dodecahedral morphology. At 16 min, however, high-index features became visible in the SEM images, confirming the shape-directing role of the iodide-induced change in Pd ion reduction rate beginning at 15 min (Figure 8E).

**Figure 8:**
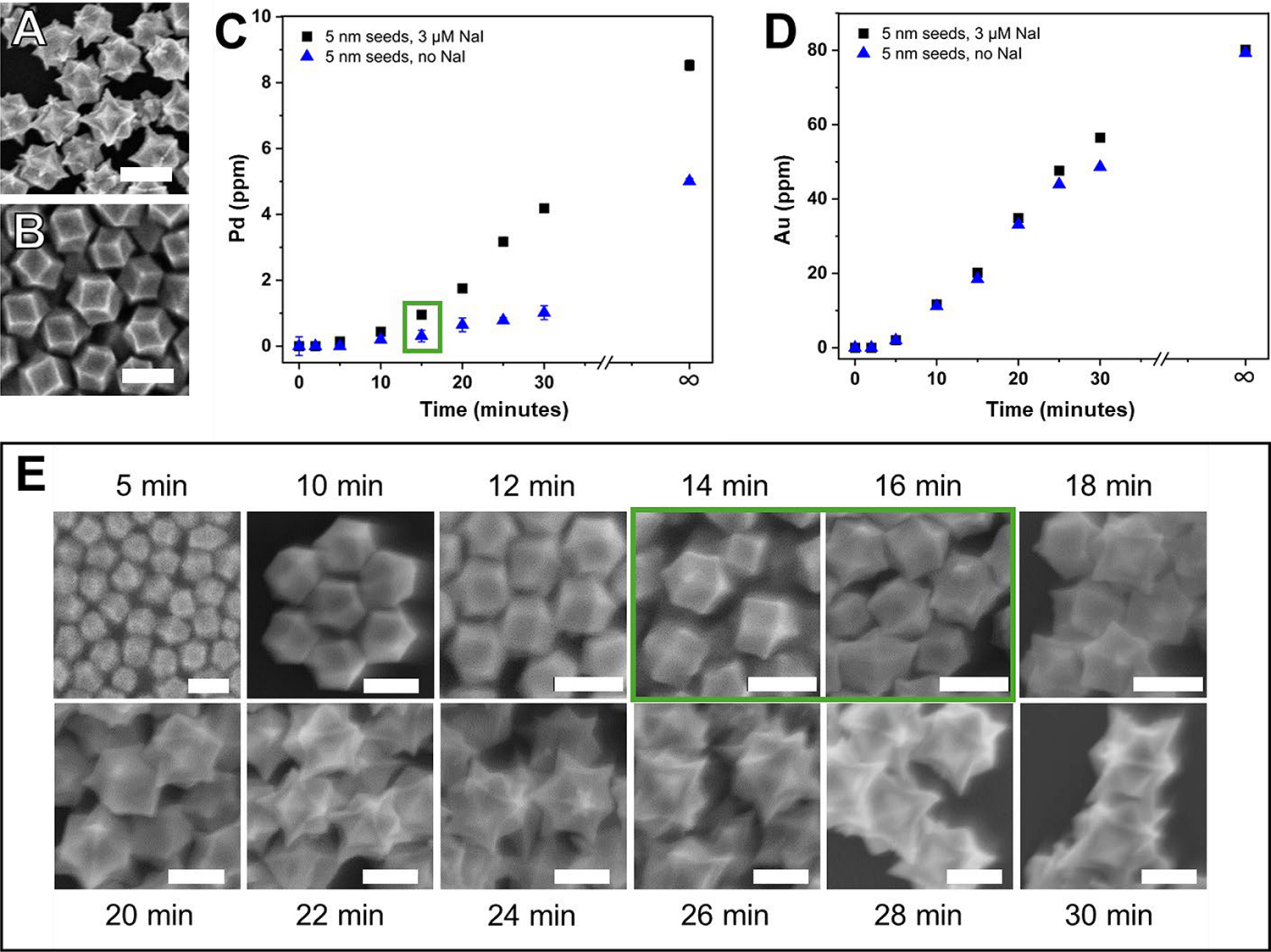
(A,B) SEM images demonstrating change in Au–Pd particle morphology with and without addition of NaI. (A) 3 μM added NaI; tetradecapod morphology. (B) No added NaI; rhombic dodecahedral morphology. (C,D) ICP-OES kinetics data showing the reduction of (B) Pd and (C) Au ions over the course of bimetallic nanoparticle growth. Data are shown for particles synthesized with and without NaI. (E) Point-in-time SEM images of the formation of Au–Pd tetradecapod particles, obtained by quenching aliquots of reactions with BSPP. Green boxes highlight the correlation between (C) the difference in Pd ion reduction rate at 15 min for syntheses with and without iodide and (E) the transition from rhombic dodecahedral to high-index proto-tetradecapod morphology at 14–16 min. Scale bars: 200 nm. Adapted with permission from ref 60. Copyright 2017 Wiley-VCH.

#### Nuclear Magnetic Resonance

Although NMR spectroscopy is frequently used for kinetic characterization and mechanism determination (as well as simple product identification and yield quantification) by synthetic chemists focusing on small molecules, metal–organic frameworks, and polymer materials, its use in the metal nanoparticle synthesis community has been far more limited. NMR spectroscopy is most commonly employed for analyzing organic reagents used during synthesis^39,102^ and probing properties of ligands at the surface of nanoparticles.^103–108^ Interpretation of NMR spectra for even simple organic molecules in solutions containing metal nanoparticles is made complex by resonance frequency shifts due to the hyperfine coupling between the nuclei of interest and the conduction band electrons at the metal nanoparticle surface (Knight shift).^109^ Additionally, probing any species appended to a metal nanoparticle via NMR will generally result in peak broadening (a powder-type pattern, similar to those seen in solid-state NMR).^110^ These effects make the assignment of small peaks and examination of more subtle changes in chemical shift (δ) complicated for some organic species of interest in nanoparticle growth solutions. Additionally, concentrations of many species of interest (such as chemical reducing agents) in nanoparticle growth solutions are often multiple orders of magnitude lower than the concentrations of other organics (surfactants, polymers), complicating peak resolution for ^1^H NMR and ^13^C NMR techniques especially. Furthermore, the most common nuclei of interest for solution-phase NMR (^1^H NMR, ^13^C NMR, ^15^N NMR, ^17^O NMR, ^19^F-NMR, and ^31^P NMR) are generally not applicable techniques for probing many of the inorganic species of interest (Cl ^–^, Br ^–^, I ^–^, transition metals) in metal nanoparticle growth solutions.

Despite these challenges, some time-resolved NMR investigations of noble metal nanoparticle growth solutions have been conducted with a focus on tracking organic ligands and reagents in solution with the growing particles. In one example, Xue et al. followed the citrate oxidation and decomposition pathway via ^1^H NMR as it reduced Ag^+^ to triangular Ag nanoprisms.^91^ In another example of the time-resolved study of citrate, ^1^H NMR was used to demonstrate the relationship between pH and final particle sizes/size heterogeneity for citrate-capped Au nanoparticles.^111^ The degree of protonation of citrate was found to determine the rate of degradation into another reducing agent (dicarboxyacetone), which in turn determined Au­(III) reduction kinetics and thus particle size.^111^ A further example used ^1^H NMR and ^13^C NMR to understand the degradation of ascorbic acid under alkaline conditions and its effects on Ag and Au nanoparticle growth.^112^ High-resolution ^1^H NMR has also been utilized to determine the speciation and role of tetrakis­(hydroxymethyl)­phosphonium chloride (THPC) and its derivatives in the reduction of metal ions and stabilization of Au, Pt, and Au–Pt alloy nanoparticles during growth.^113^ Other NMR studies have used metal-bound organic ligands to probe mechanisms or dynamics of nanoparticle growth.^114,115^

The NMR study of metal nuclei for characterizing metal nanoparticle growth is also challenging. While Au nanoparticles are otherwise generally well-characterized compared to other metals, probing ^197^Au via NMR is difficult due to its high quadrupole moment, though solid-state techniques for bulk ^197^Au-NMR have been developed.^116^ The ^107^Ag and ^109^Ag nuclei do not suffer from this issue, but have hours-long thermal relaxation (*T*_1_) times, making acquisition of large data sets time-prohibitive.^117^ Additionally, the gyromagnetic ratio of both Ag isotopes is low, leading to poor detection sensitivity and consequently the need for a different, specialized probe.^115,117^ Coupling Au or Ag to heteroatoms has had some success in nanoparticle characterization,^118,119^ and this approach has even been used to monitor the conversion of Ag­(I) into Ag nanoclusters and ∼30 nm Ag nanoparticles.^120^

In contrast to Au and Ag, ^195^Pt has advantageous NMR properties and has been studied extensively during nanoparticle growth via NMR in comparison to other metal nuclei. In early work, Pellechia et al. used ^195^Pt-NMR to track the kinetics of ligand exchange between a Pt­(II) salt and poly­(amidoamine) (PAMAM) dendrimers during a PAMAM-mediated Pt particle synthesis.^121^ These NMR spectra were used to determine the relationship between the PAMAM structure and Pt­(II) speciation and reactivity, with consequences for Pt particle growth. Straney et al. further examined Pt­(IV) and its pH-dependent reduction onto Au nanoprism substrates via ^195^Pt-NMR.^122^ This work correlated information about the chemical environment of the growth solution and Pt­(IV) speciation with mechanisms of Au–Pt interaction and, ultimately, the resulting bimetallic nanoparticle morphology—Au@Pt core–shell with Pt pendant “islands” vs hollow frame nanostructures (Figure 9A–C). This information was then coupled with time-resolved electron microscopy (Figure 9D–G) and UV–vis studies (Figure 9H) to gain an understanding of the formation kinetics of the Pt islands.

**Figure 9:**
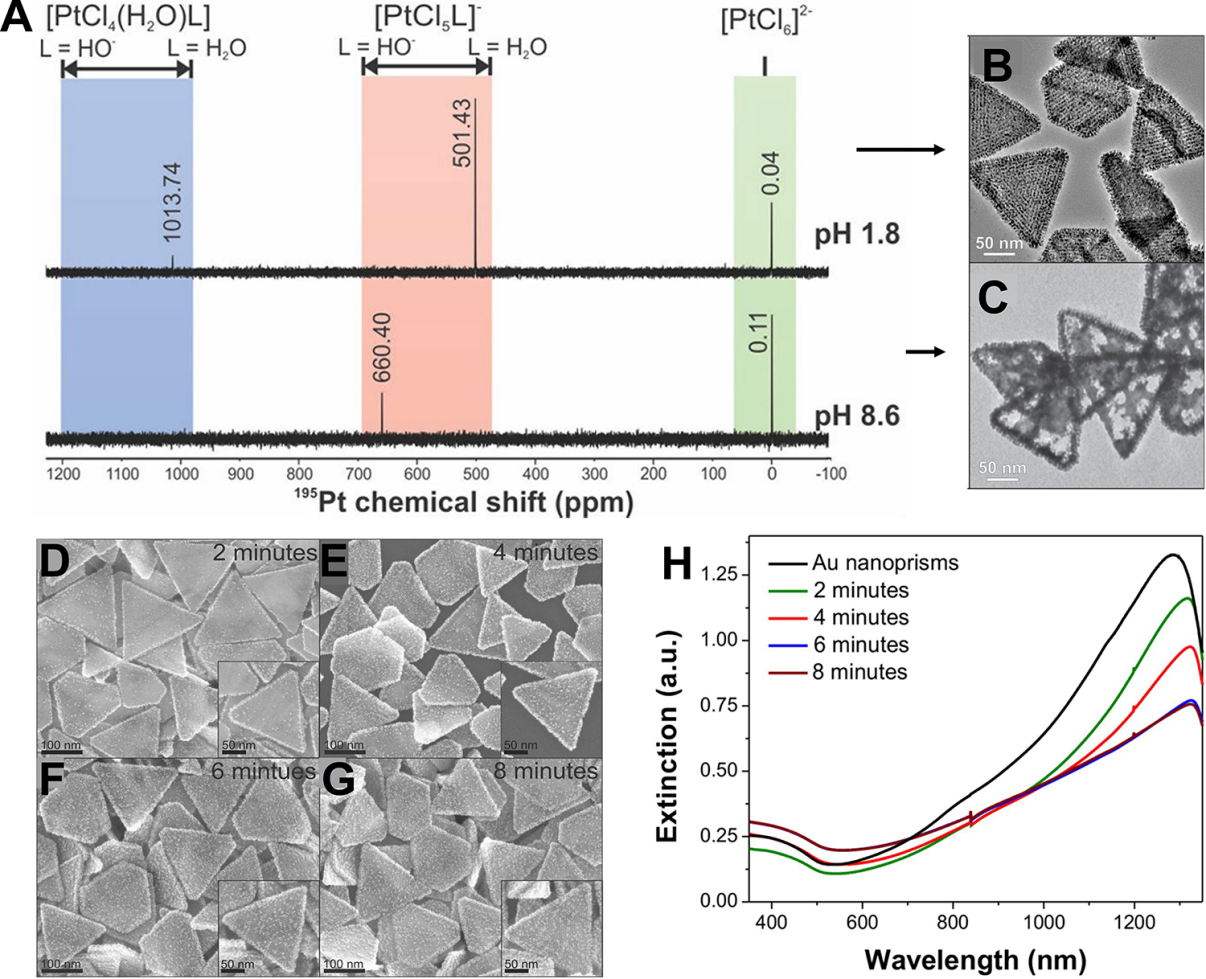
(A) ^195^Pt NMR analysis of H_2_PtCl_6_ (Pt precursor) speciation as a function of precursor solution pH. (B) TEM images of Pt deposition on Au nanoprism substrates at Pt precursor solution pH values of (B) 1.8 and (C) 8.6. Scale bars: 50 nm. (D–G) Point-in-time SEM images of Au nanoprism substrates indicating the extent of Pt deposition at (D) 2 min, (E) 4 min, (F) 6 min, and (G) 8 min after addition of H_2_PtCl_6_ to the reaction solution, with higher magnification image insets at the lower right of each panel. Larger image scale bars: 100 nm; inset scale bars: 50 nm. (H) Point-in-time UV–vis–NIR spectra corresponding to the SEM images. Panels A and C–H adapted with permission from ref 122. Copyright 2014 American Chemical Society. Panel B adapted with permission from ref 115. Copyright 2015 American Chemical Society.

### 3.2 Present Challenges in Understanding Metal Nanoparticle Growth Chemistry

Gaining a time-resolved understanding of the metal nanoparticle growth process requires careful selection of characterization techniques (Table 1). Point-in-time TEM and SEM are ideal for characterizing nanoparticle morphology throughout the growth process, but do not directly reveal anything about the chemical environment within a nanoparticle growth solution. For in situ TEM techniques, care must be taken to ensure that any changes observed are not the result of beam damage to the sample or beam-induced reactions.^67–69^ Often, additional experimentation involving variation of more individual parameters or the introduction of other characterization techniques is necessary to correlate a physical change observed through electron microscopy with a change in chemical environment. Electron microscopy also captures a small subset of the overall ensemble of nanoparticles, which is particularly important to consider in single particle measurements such as those conducted via in situ TEM.

**Table 1:** Techniques for the Time-Resolved Analysis of Nanoparticle Growth

technique	elements probed	size resolution	time resolution	instrument cost	typical location
OCP	all	N/A	0.1 s	low	in lab
chronopotentiometry/chronoamperometry	all	N/A	0.1 s	low	in lab
TEM	all	0.1 nm	1–5 min (unless in situ)	high	shared facility
SEM	all	1–10 nm	1–5 min	high	shared facility
UV–vis	Au, Ag, Cu; Pd precursors	∼4 nm	10 s–1 min	low/medium	in lab
ICP-AES/MS	all	N/A	10–15 s (quenching speed)	medium/high	in lab or shared facility
NMR	H, C, N, O, P, Pt	smaller = easier	element-dependent	medium/high	shared facility

While UV–vis is an inexpensive and simple technique for obtaining time-resolved data in nanoparticle growth, unless a previously very well-characterized particle shape is studied, the time-resolved UV–vis studies need to be paired with electron microscopy to facilitate the assignment of an LSPR peak to a specific nanoparticle shape, and this approach mainly allows for the qualitative interpretation of the reaction data. Size, shape, truncation, and aspect ratio are all known to change the LSPR of a nanoparticle and, in most growth processes, multiple of these variables are changed simultaneously, leading to a convolution of causes for a change in the LSPR extinction spectrum. This complicates the linear association of a change in the UV–vis peak with a rate of nanoparticle growth, limiting the qualitative and especially quantitative interpretation of time-resolved UV–vis spectra for plasmonic nanomaterials. While qualitative kinetic data have been obtained from measured time-resolved data of both UV–vis methods discussed here, this is restricted to synthetic systems that meet specific requirements and is not fully generalizable.^83,84,93^

Point-in-time ICP-OES/MS measurements are powerful for tracking metal ion reduction over time. Their main drawback is the time-consuming nature of sample preparation (a full day is required to prepare the samples necessary to probe a 1 h synthesis reaction) and the limited time resolution at the beginning of the growth reaction. Unlike many other commonly used techniques, they do probe a chemical process in the nanoparticle growth solution (metal ion reduction), rather than only physical changes in particles themselves. However, since these measurements only provide information about metal ion reduction rate (concentration of either metal ions in solution or reduced metal over time), they must be correlated with additional information about growth solution composition and/or nanoparticle morphology to be predictive of or descriptive about nanoparticle growth principles.

Like UV–vis spectroscopy, NMR spectroscopy can be a powerful method for probing metal nanoparticle growth and the fate of individual small molecules in a nanoparticle growth solution. However, the complexities of NMR with metal nanoparticles in the sample and/or NMR of metal nuclei, both in experimentation and interpretation, are practically limiting. On top of the challenges of interpreting spectra containing metal nanoparticles, experimental design for in situ NMR studies of nanoparticle growth can be challenging for other reasons. Standard NMR tube sizes hold much lower solution volumes than those used for most nanoparticle growth solutions, which can change important factors such as diffusion. Furthermore, the time resolution available at the beginning of a growth reaction is limited by the time necessary to take a sufficient number of NMR scans for an appropriate signal-to-noise ratio. For these reasons, adoption of NMR as a time-resolved technique for growth studies (rather than for other uses, such as examining ligand shells on metal nanoparticles following growth) by the research community has remained fairly limited despite the technique’s flexibility.

### 3.3 Open-Circuit Potential Measurements Provide In Situ Real-Time Chemical Insights

OCP measurements offer an additional option for time-resolved characterization of nanoparticle growth mechanisms that can address challenges raised by existing analysis approaches. While efforts to fully understand the contribution of individual processes and synthetic conditions to the mixed solution potential are ongoing, progress has been made in identifying the underlying chemical origins of the overall measured mixed potential. For example, tuning the ratio of reducing agent concentration to metal ion concentration is a facile way to control reduction rate in colloidal synthesis—a higher ratio of chemical reducing agent to metal ions leads to a stronger reducing environment and faster metal ion reduction. In our synthesis of Pd nanoparticles in BioUltra A CTAB, when [AA] was held constant and [Pd^2+^] was decreased, products shifted from terraced cube (TC) nanoparticles (0.38 mM Pd^2+^) to THH (0.24 mM Pd^2+^) to CC (0.15 mM Pd^2+^) (Figure 10A–C).^39^ These shifts in Pd precursor concentration—phenomenologically associated with changes in growth rate and therefore formation of different kinetic products—corresponded with changes in the measured OCP. The OCP measurement of TC growth was consistently shifted ∼0.05 V more positive (more weakly reducing) than the OCP measurement of THH growth, which was in turn shifted ∼0.06 V more positive (more weakly reducing) than the OCP measurement of CC growth (Figure 10D). These data suggest a relationship between the reducing agent and metal ion concentrations (or their relative ratio) and the kinetics of nanoparticle growth, which can in turn be observed in the strength of the reducing environment measured by the OCP method.

**Figure 10:**
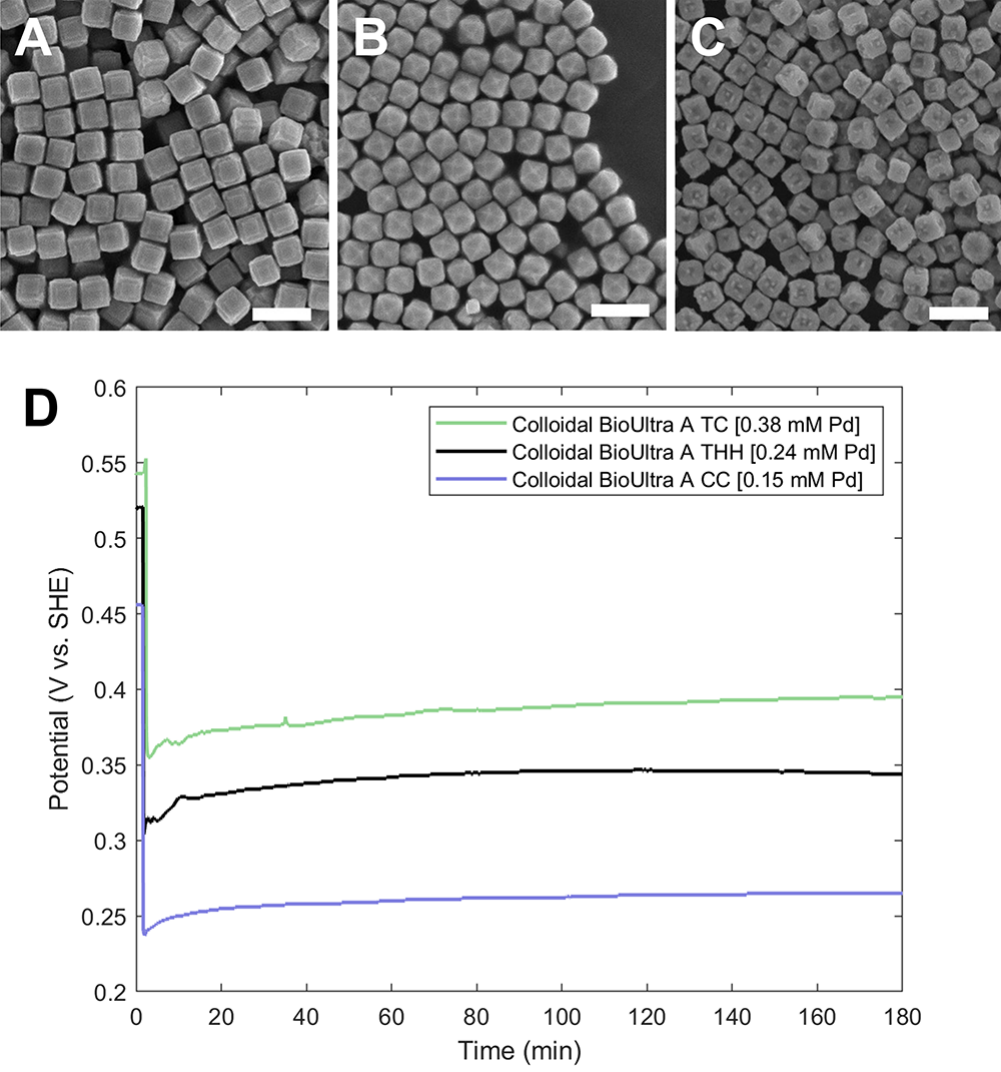
(A–C) SEM images of Pd nanoparticle shape as a function of Pd^2+^ concentration: (A) 400, (B) 250, and (C) 150 μL of 10 mM Na_2_PdCl_4_. These volumes correspond to overall concentrations of (A) 0.38, (B) 0.24, and (C) 0.15 mM of Pd^2+^. Scale bars: 300 nm. (D) OCP measurements of otherwise identical growth solutions containing 0.38 mM, 0.24 mM, and 0.15 mM of Pd^2+^. Adapted with permission under a Creative Commons CC BY-NC 3.0 license from ref 39. Copyright 2024 Royal Society of Chemistry.

The time resolution of OCP measurements is limited by the time resolution of the potentiostat instrument, not the speed of sample preparation by the researcher, leading to better capture of processes such as homogeneous nucleation of seed-like particles at the beginning of a reaction. The information provided is real-time (and can be observed in real time, without requiring any postprocessing), which has additional distinct advantages. These include greater speed of data acquisition, since no time is spent on sample preparation; elimination of concerns about appropriate methods for quenching growth for point-in-time samples; and the ability to respond to the measurement during growth if desired (i.e., adding a relevant concentration of shape-directing additive as a response to the observed solution potential following initiation of a growth reaction). Furthermore, the development of methods to report the overall chemical growth environment of the nanoparticle synthesis and the rate of the reaction over time is especially beneficial since much of nanoparticle synthesis is kinetically controlled.^4,85,123^

The in situ nature of the OCP measurement method allows for increased confidence in the relevance of the measurement to an identical “unobserved” growth reaction. This eliminates possible changes in variables such as mass transport due to changes in solution volume necessary to prepare reactions for techniques such as in situ NMR, UV–vis, or in situ TEM. It also eliminates concerns that the preparation method of samples for measurement with ex situ time-resolved techniques could be a confounding factor—for example, the roles of flocculation and particle aggregation in creating nonhomogenous distributions of particles in solution over time, which can present an issue for preparation of point-in-time ICP-OES/MS and electron microscopy samples. Finally, it is nondestructive, allowing for analysis with a secondary technique (i.e., electron microscopy) following the OCP measurement. The ability to take in situ OCP measurements without significant effects on the growth processes of noble metal nanoparticles can provide a richer understanding of nanoparticle growth chemistry, which is necessary to further build synthetic understanding.

Thus, far, we have studied only monometallic particle growth using the OCP method. The mixed potential nature of OCP method presents both challenges and possible opportunities for studying multimetallic nanoparticle growth. On one hand, the mixed potential nature of measurements means that the reduction of one metal vs another cannot be explicitly separated, as they can be with other electroanalytical techniques such as cyclic voltammetry. On the other hand, measurement of the mixed solution potential provides a direct opportunity to study multimetallic growth where one metal influences or changes the reduction pathway of another.

OCP measurements of colloidal systems have enabled successful translation of colloidal synthesis conditions to “colloidal-inspired” electrochemical deposition onto an electrode surface. (Fundamentals of metal nanoparticle electrodeposition will be discussed further in Section 4.2). The approach for translating colloidal synthesis to electrodeposition conditions involves taking OCP measurements of the colloidal growth reaction, then empirically identifying an applied current that will replicate that potential over time when applied to the same growth solution in the absence of a chemical reducing agent (Figure 11A). The applied current, rather than a chemical reducing agent, supplies electrons to the metal ion reduction reaction. Applying a potential similar to the OCP potential has been found to be less reliable for the replication of colloidal synthesis via electrodeposition, but should also be a viable approach with further study. When the measured potential during a “colloidal-inspired” electrodeposition is in good agreement with the colloidal OCP measurement, the electrodeposited nanoparticles are the same shape as those synthesized colloidally.^50,124^

**Figure 11:**
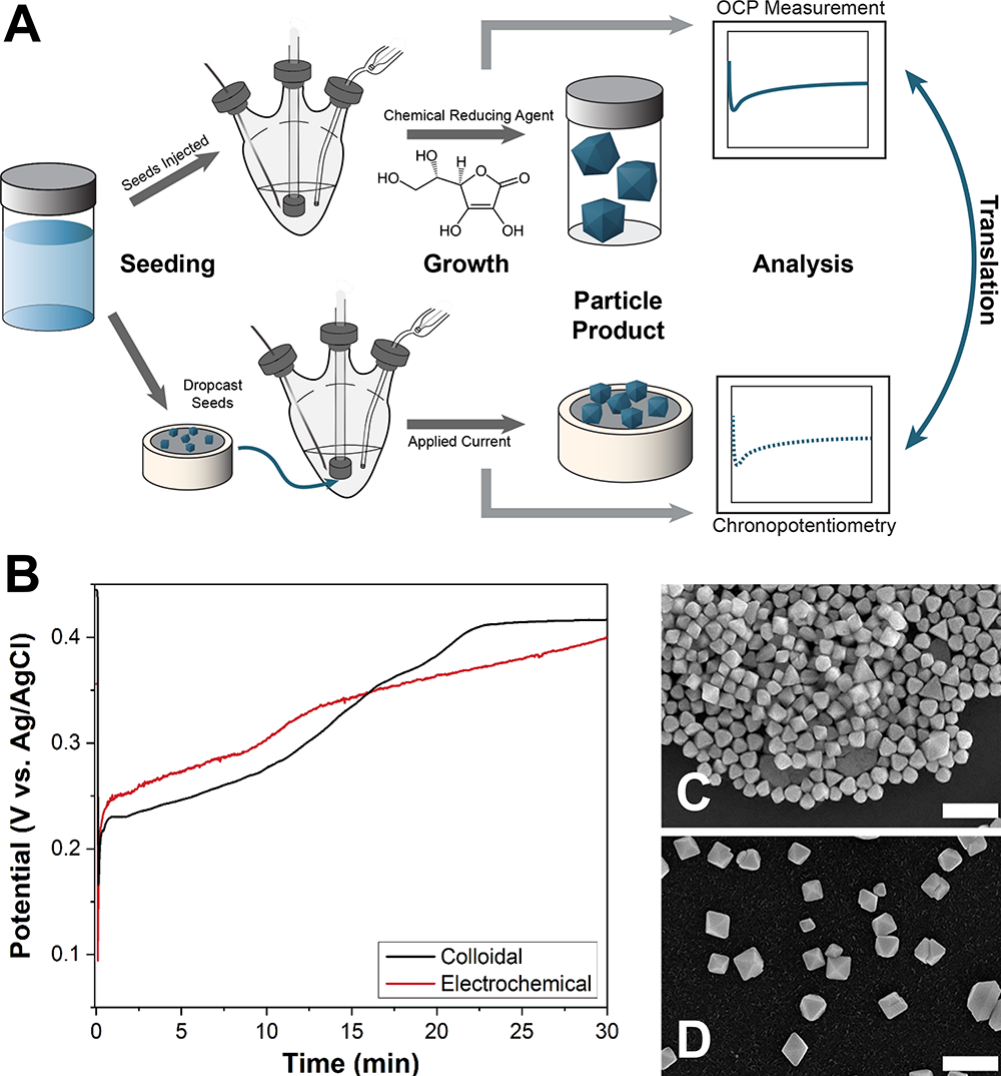
(A) Schematic of a colloidal synthesis with corresponding OCP measurement (top) and its subsequent translation to electrodeposition (bottom). (B) Comparison between time-resolved potential profiles for colloidal synthesis (OCP measurement) and electrodeposition (chronopotentiometry measurement) of Pd octahedra. (C,D) SEM images of (C) colloidally synthesized and (D) electrodeposited Pd octahedra. The electrodeposition synthesis was conducted using a galvanodynamic current ramp from −20.4 to −2.55 μA/cm^2^ over 30 min. Scale bars: 200 nm. Panels B–D adapted with permission from ref 50. Copyright 2020 American Chemical Society.

We initially validated this colloidal-to-electrodeposition translation approach with the successful translation of the colloidal chemical synthesis of Pd cubes and octahedra to electrodeposition on an electrode surface.^50^ The mixed solution potential of both colloidal growth reactions sloped upward over the course of growth, which was attributed to the oxidation of the chemical reducing agent, which was not in significant excess. To match this, a current ramp that became less strongly reducing over time was programmed for the electrodeposition. Application of this current ramp led to a potential profile over the course of the electrodeposition reaction that tracked well with the OCP measurements (Figure 11B) and matching colloidal and electrodeposited nanoparticle morphologies were subsequently confirmed for both shapes by SEM (Figure 11C,D). Recently, the development of an electrodeposition method inspired by our colloidal synthesis of high-index Pd nanoparticles with shapes controlled by acetone and iodide impurities in CTAB provided further evidence for translatability between solution growth and electrodeposition.^39^ Overall, the correspondence between colloidal synthesis and electrodeposition suggests possible opportunities for using electrodeposition to directly accelerate synthesis discovery, rather than only using colloidal synthesis to inform synthesis design for electrodeposition.

## 4 Synthesis Design

### 4.1 State of the Field

As described earlier, the present approach for synthesis development for new nanoparticle architectures and/or compositions often involves a combinatorial, iterative process. Consequently, many colloidal synthetic routes belong to the same complex family tree, tracing back to examples such as the Turkevich synthesis for citrate-capped Au nanoparticles^125^ and the Au nanorods developed independently by the Murphy and El-Sayed research groups.^20,126^ Developing novel synthetic approaches without iterating on the conditions of a previously published synthesis remains challenging, which in turn limits the ability to develop syntheses for certain desired architectures, such as shape-controlled non-noble metal particles, multimetallic particles, or particles with unusual high-index facet structures. Combinatorial or stepwise screening approaches are generally time-consuming when not guided by chemical design principles. Additionally, while iterating on previous work has thus far been very successful both in generating syntheses for new particle morphologies and in building a framework for physical and structural understanding of nanoparticle growth processes, the discovery of new synthetic routes for low-index and other easier-to-attain products is gradually being exhausted.

Meeting demands for the development of new synthetic conditions involves confronting the limitations of colloidal nanoparticle synthesis with a chemical reducing agent. One major challenge is the relatively small library of chemical reducing agents—l-ascorbic acid, sodium borohydride, citric acid, hydroquinone, hydroxylamine, hydrazine, polyols, and others—commonly used in colloidal nanoparticle synthesis.^127^ The limited number of chemical reductants available essentially quantizes the available reducing strengths/potentials for metal ion reduction, in turn shrinking the scope of available chemical syntheses.

### 4.2 Electrodeposition Approaches for Noble Metal Nanoparticle Synthesis

Like colloidal synthesis, electrodeposition yields control over the size, shape, and other structural characteristics of metal nanoparticles.^128^ Rather than adding a chemical reducing agent as in colloidal synthesis, the Fermi level of the working electrode is changed. This allows for the regulation of the electron supply to the nanoparticle growth reaction through experimental control of the potential or current density applied to the working electrode by a potentiostat. This enables precise control of the kinetics of metal ion reduction on the working electrode and allows for more accurate manipulation of the final product morphology. Fine control of the applied potential or current density can adjust the rates of the redox reactions, while temperature and reagent concentrations further influence the growth and shape of the nanostructures.^128^ This overall provides access to a far greater number of different reducing environments available for designing new syntheses, in contrast to the more limited possibilities of chemical reduction.

Electrodeposition as a standalone method, without parallel insights from colloidal syntheses, has been used to synthesize a wide range of materials, including thermodynamically favorable nanostructures—such as nanowires, cubes, octahedra, and nanosheets—of common metals (Au, Pd, Pt, and Cu).^128^ Electrodeposition syntheses of shaped multimetallic nanostructures—Pt-rare earth metal alloys and iron–nickel (Fe–Ni) alloys—have also been reported, although these are rarer.^128^ Electrodeposition is generally classified into two main categories: the galvanostatic method and the potentiostatic method. In the galvanostatic method (chronopotentiometry), the current is kept constant while the potential at the electrode surface is allowed to vary.^51,129^ This method is the closest analogy to colloidal chemical synthesis.^50^ In contrast, the potentiostatic method (chronoamperometry) involves applying a constant potential for a certain duration to electrodeposit metal nanoparticles.^51,129^ This method differs from colloidal synthesis, as the solution potential in colloidal synthesis will naturally increase over the course of colloidal synthesis as more of the reductant is oxidized.^50^ In contrast, potentiostatic electrodeposition will typically involve application of an increasingly strong current over time to maintain the same potential, causing different effects in later stages of particle growth.^130^ Galvanodynamic and potentiodynamic methods, involving applied current or applied potential ramps, respectively, are also occasionally used.^50^

Just as seeds are used to catalyze metal ion reduction and control nanoparticle growth in colloidal synthesis, the use of a nucleation or seeding step can be employed in electrodeposition. Single-step, “conventional” electrodeposition methods, which lack a separate seeding step, result in ongoing nucleation during nanoparticle growth, which often results in polydispersity and affects the shape and size of the newly formed particles.^130^ This issue can be addressed by separating the nucleation and growth steps. Uniform nuclei or seeds can be generated by applying a large initial overpotential for a short period. Following this, slow growth can be achieved by carefully controlling the potential or current density, which prevents further nucleation and allows for controlled growth onto the seeds.

Recently, electrophoretic deposition has been utilized to immobilize citrate-capped Au nanoclusters uniformly on electrode surfaces via the electrochemical oxidation of hydrogen peroxide or hydroquinone.^131,132^ Electrophoretic deposition involves two processes: migration of charged particles present in the solution/suspension toward the electrode surface under an applied potential, followed by the accumulation and deposition of the particles onto the electrode surface. This method has been used to study the electrochemical growth kinetics of Au nanoparticles as a function of Au nanocluster size.^133^ Other examples of seeded electrochemical syntheses in the literature have been conducted using double-step chronopotentiometry experiments, such as the synthesis of high-index faceted Au nanocrystals in deep eutectic solvents by Wei et al.^134^ Following application of the same nucleation overpotential, variation of the growth potentials (*E*_growth_) between −0.45 and −0.6 V led to a shape evolution from Au concave trisoctahedra (TOH), to stellated concave TOH, to concave cubes, to nanocubes.

Recently, pulsed electrodeposition methods have been employed to control the growth of nanoparticles, enabling the synthesis of metal nanoparticles with an expanded range of shapes.^128,129^ The pulsed electrodeposition process consists of an initial step to relax the compositional double layer, followed by repeated, alternating anodic and cathodic pulses of either current density or potential to produce uniquely shaped, often high-index nanoparticles.^129^ Pulsed techniques are unique to electrodeposition, as the rapid cycling of oxidizing and reducing conditions is not presently feasible for colloidal nanoparticle synthesis.^128^

One common pulsed electrodeposition technique is the square wave potential method, which can generate metal nanoparticles with complex structures by oscillating between an oxidizing upper potential (*E*_U_) and a reducing lower potential (*E*_L_) (Figure 12A).^135^ Examples of high-index structures synthesized using a square wave potential include Pt tetrahexahedra^136^ and Pd tetrahexahedra (Figure 12B).^137^ Shape evolution of Pd nanocrystals electrodeposited with a square wave potential from cubes to truncated octahedra has also been demonstrated, mediated by the concentration of the PdCl_2_ precursor salt.^135^ Additional parameter space is opened through variation of either *E*_U_ or *E*_L_ while holding the other potential constant. For example, Wei et al. electrochemically synthesized faceted Pd nanocrystals using a square-wave method in choline chloride-urea based deep eutectic solvent.^138^ By keeping *E*_L_ at −0.40 V and increasing *E*_U_ progressively from – 0.05 to 0.05 V vs a Pt quasi-reference counter electrode, the shape of the Pd nanocrystals was changed from a mixture of octahedra and icosahedra to concave–disdyakis triacontahedra.

**Figure 12:**
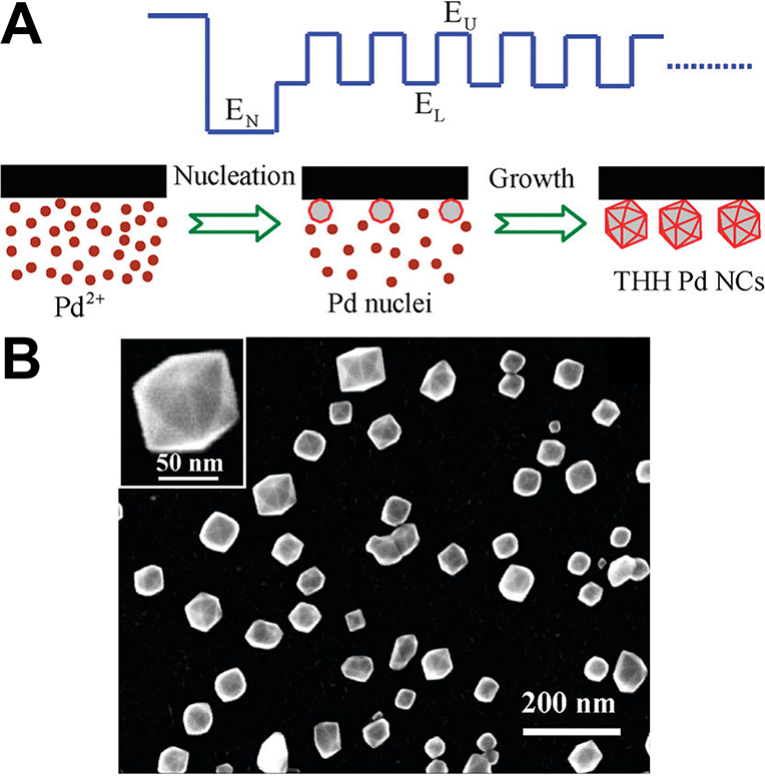
(A) Schematic of the square-wave potential electrodeposition method for synthesis of Pd THH nanoparticles. (B) SEM image of Pd THH Pd nanoparticles and inset of high-magnification SEM image showing a single Pd THH. Larger image scale bar: 200 nm; inset scale bar: 50 nm. Adapted with permission from ref 137. Copyright 2010 American Chemical Society.

The added flexibility in choosing an applied current or potential that is not fixed by the selection of available chemical reducing agents, as well as options for pulsing of redox conditions and the ability to change the growth environment over multiple steps, make electrodeposition methods promising for the synthesis of all metal nanoparticles. They are especially poised for further development of synthetic conditions that are challenging to achieve by colloidal synthesis. These include the development of nonpolyol thermal syntheses for shaped Ag nanoparticles and conditions for the shape control of Pt. Pt represents an especially interesting case, as there are presently few reports of successful colloidal faceted shape control (even for conditions similar to those which produce shaped Pd nanoparticles), yet shape control of Pt nanoparticles has been achieved through electrodeposition repeatedly.^136,139–142^ The increased abundance of electrodeposition syntheses compared to colloidal syntheses for Pt underlines the opportunities that emerge from the wider parameter space of reducing environments available in electrodeposition.

The expanded parameter space afforded by electrodeposition also invites new research into techniques for the synthesis of nonplatinum group nanostructures, particularly Cu, Ni, and cobalt (Co), for which there are far fewer well-established colloidal synthesis routes to shaped nanoparticles.^143^ As previously discussed, the majority of colloidal syntheses for noble metals have been developed through an iterative approach starting from reported syntheses for Au and Pd; however, this approach is likely not feasible for the development of non-noble metal nanoparticles synthesis, as the synthetic conditions for these materials require the use of stronger reducing environments or higher reaction temperatures in colloidal synthesis than are typical for noble metal nanoparticles.^11,14,143^ Electrodeposition could present an advantage over colloidal chemical synthesis in this case. The added flexibility in reducing environment design provided by electrodeposition methods may also aid in the development of multimetallic nanoparticle syntheses. Shape control of alloy nanoparticles remains a challenge due to the need to either closely match the reduction potentials of all metals or to make the reducing environment (applied current/potential or chemical reducing environment) so strong that the difference between metal ion reduction rates is minimized.^128^ The ability to control both chemical and electrochemical parameters simultaneously in electrodeposition synthesis may allow for better achievement of these synthetic needs.

Additionally, the omission of chemical reducing agents from the electrodeposition growth solution in turn allows for omission of acid or base additives used to mediate the strength of the chemical reductant, as demonstrated by the adaptation of a colloidal synthesis for corrugated Pd nanoparticles to an electrodeposition synthesis.^50^ The ability to decouple pH from the applied reducing environment in electrodeposition may provide additional options for promoting the formation of metallic vs metal oxide nanostructures, as copper oxide formation, for instance, is known to be pH-dependent.^144^

With respect to mechanistic studies of nanoparticle growth, an advantage of electrodeposition is that deposition can be easily and rapidly stopped at any time for characterization of the “intermediate” nanoparticle products. The presence of the growing nanoparticles on the electrode surface also facilitates additional characterization by complementary electroanalytical methods. For instance, linear sweep voltammetry has been used to study the electrochemical growth kinetics of Au nanoparticles with respect to the seed size.^133^ Underpotential deposition has also been used to characterize the surface area and structure of noble metal nanoparticles. This technique involves the irreversible site-specific adsorption of adatoms onto the nanoparticle surface under underpotential conditions. After adsorption, subsequent quantitative stripping of the adatoms reveals the relative amounts of different surface facets, and this approach has been used to characterize Pt spheres, cubes, octahedra, tetrahedra, and truncated octahedra.^145,146^ Unfortunately, real-time electrochemical analysis via these types of methods during the growth process is challenging, since electrochemical characterization methods such as stripping voltammetry or underpotential deposition tend to be destructive.

While electrodeposition presents unique possibilities and opens additional parameter space for the synthesis of non-noble metal nanoparticles and alloy nanoparticles, additional experimental considerations must be taken, as the available applied potential range is not unlimited in these cases. The onset of electrolyte decomposition, particularly due to the hydrogen evolution reaction (HER)—and, less importantly, the oxygen evolution reaction (OER)—in aqueous electrolytes, poses a limit to the available potential range that may be narrower than theoretical potentials of interest for reduction of non-noble metal ions. Avoidance of the HER and OER are crucial for shaped particle synthesis, as gas evolution during electrodeposition results in the formation of unfaceted nanocrystalline structures.^130^ The onset of the HER can be tuned by controlling the pH of the aqueous electrolyte if more reducing (lower) potentials are required, although an increase in pH can also concurrently increase the likelihood of metal oxide formation. Alternatively, a switch to a nonaqueous electrolyte can tune the accessible potential window, while avoiding an increased oxide formation, although precursor solubility and solvent cost need to be considered.^147^

### 4.3 Combinatorial Electrodeposition for Synthesis Discovery

A present drawback of electrodeposition compared to traditional colloidal nanoparticle synthesis is that electrodeposition is usually serial and consequently low throughput. Existing electrodeposition setups generally require a dedicated potentiostat for each experiment, limiting the number of conditions that can be screened simultaneously.^148^ The development of high-throughput electrodeposition approaches would enable the combination of the increased parameter space of electrodeposition with a high simultaneous number of experiments—traditionally a trademark of colloidal synthesis—accelerating the implementation of a combinatorial approach in electrochemical material synthesis.

Using a multichannel potentiostat known as “Legion” that is based on the dimensions of a 96-well plate with 96 independently controllable quasi-reference counter electrodes and a large single glassy carbon working electrode (Figure 13A),^149^ our research group, in collaboration with the Baker Group at Texas A&M University, recently demonstrated that it is possible to increase the throughput of electrodeposition to match that of colloidal synthesis.^148^ Proof of concept for this combinatorial electrodeposition technique was established via the implementation of a two-part synthesis discovery approach for shaped Pd nanoparticles. In the first stage, the chemical environment of the synthesis (surfactant, metal precursor concentration, etc.) was screened for promising conditions that led to the deposition of moderately well-defined polyhedral nanoparticles. In the second stage, the electrochemical parameter space (potential, constant vs square wave potential profile, deposition time) was investigated to optimize conditions for the uniform deposition of nanoparticle shapes that emerged from the first stage of screening—in this case Pd cubes (Figure 13B). Importantly, the optimized deposition parameters that resulted from the combinatorial experiment in the two-electrode cells of the Legion setup were directly translatable to a standard bulk three-electrode cell (Figure 13C,D), thereby establishing the feasibility of this increased throughput approach and adding combinatorial nanoparticle synthesis screening capabilities to the approaches accessible via electrodeposition. These combinatorial capabilities hold promise both for advancing the discovery of novel-shaped nanoparticles for the well-studied noble metals Au, Pd, and Pt by building on already established electrochemical syntheses for these materials and for accelerating the development of novel shape-selective conditions for the synthesis of non-noble metal nanoparticles.

**Figure 13:**
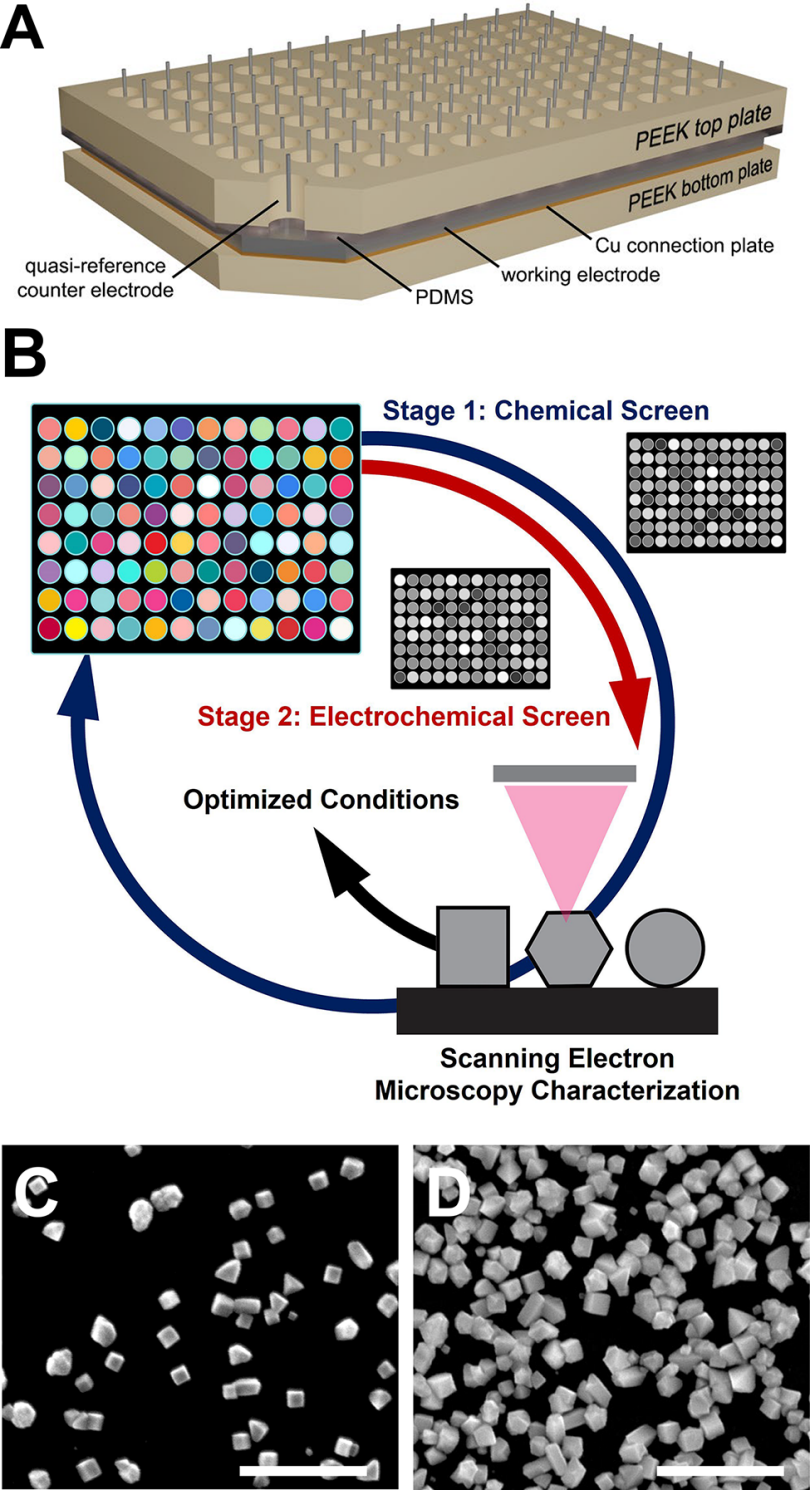
(A) Schematic illustration of the Legion 96-well plate design and electrode geometry. (B) Schematic representation of the experimental workflow for synthesis discovery and optimization via array-based parallel electrodeposition of metal nanoparticles, with separation of chemical and electrochemical screening stages. (C,D) SEM images demonstrating translation of the optimized Pd nanocube synthesis conditions identified with Legion to a bulk three-electrode cell. (C) Pd nanoparticles electrodeposited using Legion (Ag/AgO QRCE). (D) Pd nanoparticles electrodeposited using analogous conditions in a traditional bulk cell (Ag/AgCl || Pt; all deposition potentials shifted +193 mV to account for the difference in reference electrode potentials between Ag/AgCl and Ag/AgO). Scale bars: 500 nm. Adapted with permission under a Creative Commons CC BY 4.0 license from ref 148. Copyright 2024 American Chemical Society.

## 5 Outlook

Looking beyond the separate roles of electrochemistry in (1) measurement of colloidal synthesis and (2) materials discovery via electrodeposition, additional emerging opportunities exist at the interface of electrodeposition and colloidal synthesis, both for synthesis design and for mechanistic understanding. As one example, some chemical additives necessary for colloidal synthesis can be omitted entirely from synthesis via electrodeposition. For instance, surfactant- and capping agent-free synthetic routes are possible in electrodeposition, where they would be impossible due to particle aggregation for successful chemical synthesis, as are some synthetic routes with the omission of acid or base additives.^50,124^ Consequently, by taking advantage of the parallels between nanoparticle growth in colloidal synthesis and electrodeposition under well-controlled, analogous conditions, it becomes possible to directly study the roles of individual reagents in colloidal synthesis by probing the influence of their presence and absence on the outcome of electrodeposition. These insights can then be applied to better inform colloidal synthesis design. This approach to isolating the specific chemical roles of individual reagents under particular reaction conditions could be especially powerful in tandem with insights from electrochemical studies utilizing single crystal electrode measurements to understand adsorption of reagents of interest onto model metal surfaces, such as those by Wiley and co-workers examining the adsorption of halide ions and ionic surfactants onto Cu, Au, and Ag surfaces.^150–153^ The immobilization of nanoparticles on a substrate during electrochemical growth also allows for easier tracking of individual particles throughout the growth process and therefore improved understanding of particle-to-particle heterogeneity, as reported by Verma et al. in a recent study of Au electrodeposition onto colloidally prepared Au cubes, which explored the roles of CTAB and multiple applied potential conditions on final Au particle morphology.^154^

In addition, the added flexibility of electrodeposition to discover synthetic conditions outside of those accessible using common reducing agents presents a possible method of identifying reaction environments that would be successful at producing a particular shape in colloidal synthesis. Establishing ideal reducing conditions for the growth of novel shapes using electrodeposition and then translating from electrodeposition to colloidal synthesis—the reverse of the process described in Section 3.3—could be more generally instrumental in identifying chemicals to serve as novel reducing agents for materials whose synthesis is currently prohibitively challenging, such as shape-controlled syntheses for nanoparticles of non-noble transition metals. Combinatorial iteration on colloidal syntheses developed with this approach could then yield more productive discovery of new colloidal syntheses. The success of translation from electrodeposition to colloidal synthesis, for which our research group established preliminary proof of concept,^50^ may also require the use of dynamic synthetic conditions, such as the addition of reagents at specific time points during nanoparticle growth or in a controlled manner throughout growth instead of only at the beginning of the synthesis.

Finally, the process of troubleshooting irreproducibility could be streamlined by creating a data set of benchmark OCP measurements for all synthetic conditions of interest when a newly developed nanoparticle synthesis is reported, to preliminarily identify variations in synthetic parameters that induce specific changes in OCP measurements. Comparison to such a data set would then allow for even faster “informed guessing” as to how to alter the mixed potential of a nanoparticle growth solution to match a desired benchmark measurement should a synthesis by imperfectly reproducible in a different lab. Open access to such a data set would facilitate synthetic reproducibility efforts throughout the research community and would provide information that could be used to understand trends in reactivity in mechanisms across different synthetic systems. The utility and impact of this information could also be increased by using rapidly advancing tools such as large language models and machine learning both to accelerate troubleshooting of syntheses and to uncover mechanistic trends for use in establishing a comprehensive set of predictive design principles.
